# Loose social organisation of AB strain zebrafish groups in a two-patch environment

**DOI:** 10.1371/journal.pone.0206193

**Published:** 2019-02-08

**Authors:** Axel Séguret, Bertrand Collignon, Léo Cazenille, Yohann Chemtob, José Halloy

**Affiliations:** Univ. Paris Diderot, Sorbonne Paris Cité, LIED, UMR 8236, 75013, Paris, France; University Zürich, SWITZERLAND

## Abstract

We study the collective behaviour of zebrafish shoals of different numbers of individuals (1, 2, 3, 5, 7, 10 and 20 AB zebrafish *Danio rerio*) in a constraint environment composed of two identical square rooms connected by a corridor. This simple set-up is similar to a natural patchy environment. We track the positions and the identities of the fish and compute the metrics at the group and at the individual levels. First, we show that the number of fish affects the behaviour of each individual in a group, the cohesion of the groups, the preferential interactions and the transition dynamics between the two rooms. Second, during collective departures, we show that the rankings of exit correspond to the topological organisations of the fish prior to their collective departure. This spatial organisation appears in the group a few seconds before a collective departure. These results provide new evidences on the spatial organisation of the groups and the effect of the number of fish on individual and collective behaviours in a patchy environment.

## Introduction

Across the collective behaviours observed in social animals, collective movements [1–8], nest site selections [9–12] and site transitions [13] have been evidenced in many species. In this latter case, the groups face several alternatives and transit between them. The study of these transitions relies on decision-making processes and individual or collective preferences for environmental [14] or group members characteristics [3, 15–17] like leadership [18], motion [19] or behavioural traits, for example bold and shy individuals [12, 20].

Numerous animal species have been observed in different sorts of constraint setups or mazes to study collective movements from one site to another: corridor type [3, 16, 21], Y-maze [22], T-maze [23] or Plus-maze [24, 25]. Such constraint set-ups engage the animals to transit alone or in group from site to site and allow the observation of leadership [26–28], initiation of group movements [19, 26, 29], followers organisations [26], pre-departure behaviours [19, 27] and sites transitions [13, 30, 31]. In these latter cases the authors studied the transitions from one site to the other of one and two fish separated by a transparent partition (*Gasterosteus aculeatus* and *Sciaenops ocellatus*). Although such experimental procedure provided evidence of different leader/follower behaviours in fish, they prevent the fish from direct interactions between each other during the departures.

On the one hand, studies performed with groups of fish swimming together have evidenced that the group size can impact swimming behaviours with a variety of results. Several papers showed that the speed, the turning speed, the nearest neighbour distances, the milling or the alignment are affected by the number of group members [32–34]. The authors present opposite results depending on the species: increasing the group size of *Oreochromis niloticus* (330 and 905 fish), makes a stronger alignment [32], while for *Notemigonus crysoleucas* (30, 70, 150 and 300 fish) alignment decreases [34]. On the other hand, *Shelton et al*. [35] have shown that the density influenced nearest neighbour distances in *Danio rerio* when *Frommen et al*. [36] noticed that shoaling preferences might not always be influenced by a higher number of group members but also by the density and cohesiveness of the respective groups.

We focus on the collective movements between two environmental patches of different numbers of zebrafish. We have shown in a previous study that zebrafish transit without interruption from one landmark to another one in an open environment during trials of one hour [37]. Moreover, we have shown that groups of fish were swimming along the border of the tank and thus had a strong thigmotactic tendency [38]. Inspired by the experiments developed for highly dynamical groups of animals like the ants [39] or the fish [3, 16, 21] in constraint set-ups, we created a binary choice set-up able to channel the groups of zebrafish and to increase their stabilisation in the patches. Our experimental set-up is composed of two environmental patches (rooms) linked by a corridor. The geometry of the setup is designed to study collective transitions between patches allowing to quantify the group cohesion and collective decision-making. In this study, we aim at characterising the dynamics of departure during sites transitions for several group sizes (1, 2, 3, 5, 7, 10 and 20 individuals) of AB zebrafish swimming in a constraint environment. Here we consider group size as the number of fish in a group.

Zebrafish are gregarious vertebrate model organisms often used in behavioural studies [40, 41]. In the laboratory as much as in the nature, the zebrafish behave in groups [3, 42, 43]. They are native to the Indian sub-continent and live in small groups or in big shoals of several hundreds of fish depending on the region and the water or the environmental features (temperature, pH, human activity, predators, …) [15, 44–46]. Zebrafish live in a wide variability of habitats with varying structural complexities [45, 47] (from river channels, irrigation canals to beels) and we based our experimental method on the observations of fish swimming in a constraint set-up composed of two identical squared rooms (evoking patchy environments [48]) connected by a long corridor. The goal of the paper is to measure the impact of the groups of fish on the collective decision making between two identical patches. This methodology has been developed in [10].

Here, we study the collective dynamics of group transitions in zebrafish with a new type of set-up. By observing groups composed of different numbers of fish, we evaluate the influence of the number of individuals in the shoals on the structure of the group (cohesiveness, inter-individual distances) and on the sequence of exit for each collective departure. By performing trials of one hour, we could observe a large number of successive transitions.

## Results

### Group structure and number of individuals

**Fig 1:**
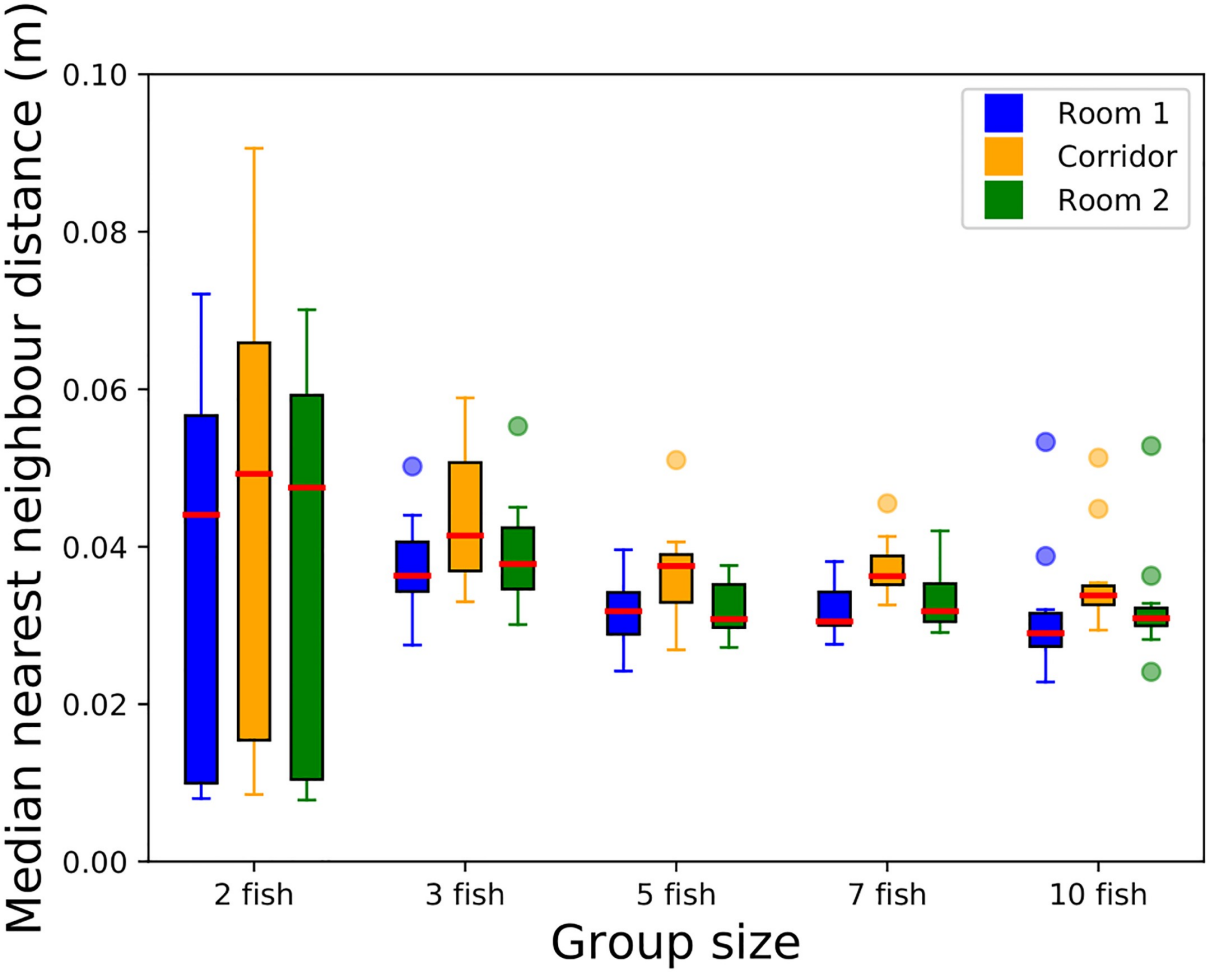
Boxplots of the medians of the nearest neighbour distance distributions for each zebrafish (blue) in the room 1, (green) in the room 2 and (yellow) in the corridor. The red line is the median. The higher the number of individuals, the lower the nearest neighbour distances between fish.

First, we studied the change of the group structure according to the location of the group and the number of fish by measuring the nearest neighbour distances for each individual. Fig 1 shows the boxplots of the medians of the nearest neighbour distance distributions for each fish in 5 shoal sizes (2, 3, 5, 7 and 10 fish). We chose to use the Nearest Neighbour Distance (NND) because we wanted to describe the shoal dynamics. If we took an average of all Inter-Individual Distance (IID), this would have been higher in larger shoals than smaller shoals because larger shoals take up more volume. Also the NND lowers the effect of the geometry of the set-up compared to IID. Thus, the boxplots for each area (rooms or corridor) and each number of individuals consist in 12 values of medians. For groups of 2 to 3 individuals, the increase of the number of individuals made the medians of the nearest neighbour distances decrease until a plateau value of approximately 4 cm. For groups of 5, 7 and 10 individuals, the medians of the nearest neighbour distances remained very close from each other.

We compared with a Two-way ANOVA the distributions of the medians of the nearest neighbour distances for each fish focusing on each area (room 1, room 2 and corridor) or each number of individuals. The test shows that there is an effect of the number of individuals on the medians of the nearest neighbour distances (*p*–*value* < 0.005, *F* = 3.87, *MS* = 0.00092 and *df* = 4). However, it does not show any significant effect of the type of the area—Room 1, Room 2 or Corridor—(*p* − *value* > 0.1, *F* = 1.96, *MS* = 0.00047 and *df* = 2) nor of an interaction between the number of individuals and the type of the area (*p* − *value* > 0.5, *F* = 0.15, *MS* = 0.00004 and *df* = 8).

### Oscillations and collective departures

**Fig 2:**
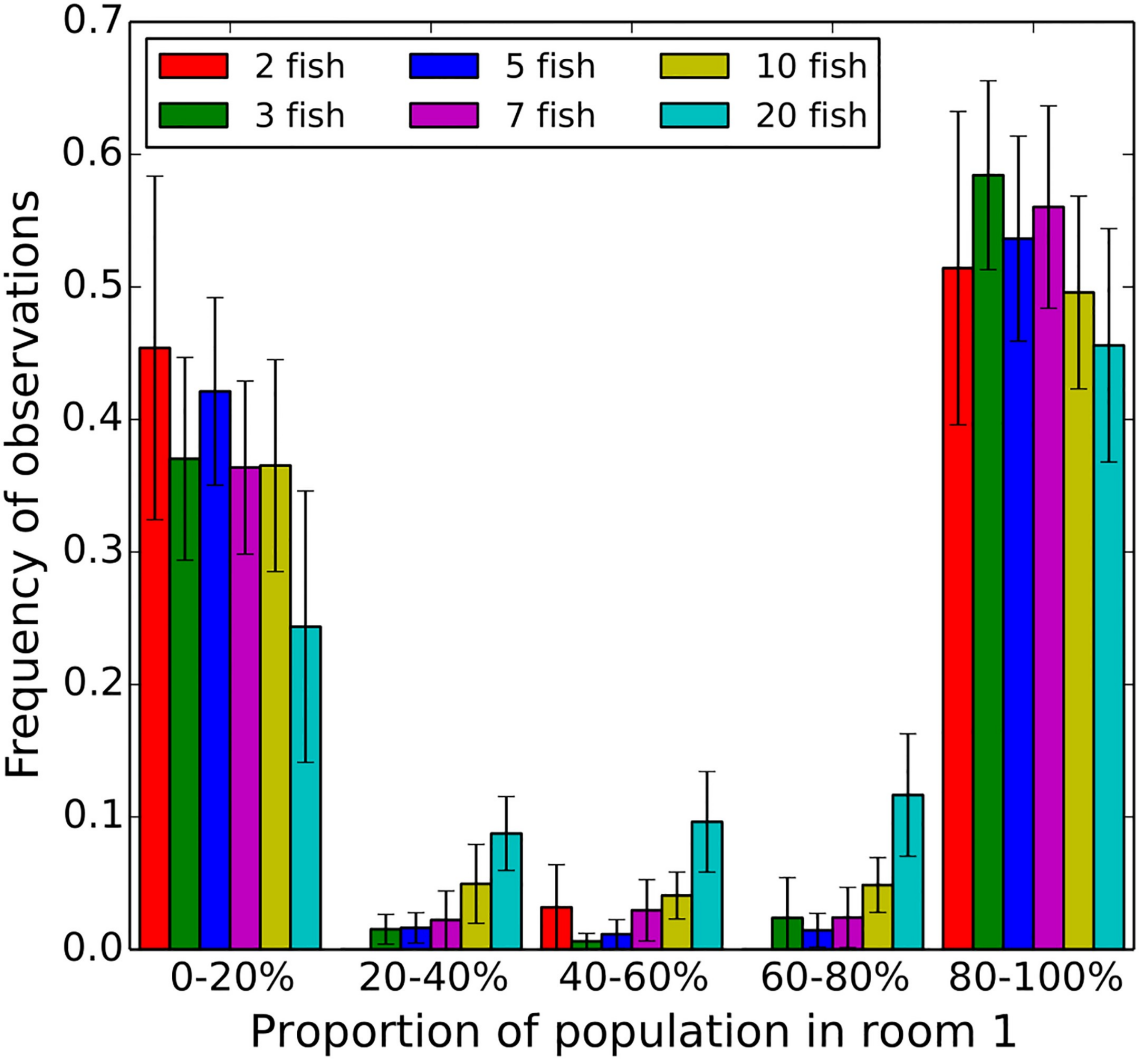
Frequency of the proportion of the whole group in room 1. Almost 35% of the time, 0 to 20% of the whole group is present in the room 1 when almost 50% of the times, 80 to 100% of the whole group is in the room 1. Focusing on more equal repartition of the fish between the rooms (40 to 60% of the whole group), larger groups lead to higher frequency of group splitting.

Then, we characterised the collective behaviours of the fish. In particular, we focused our investigation on the oscillations between both rooms and the dynamics of the collective departures of the groups. First, we studied the repartition of the fish among the two rooms. Approximately 70% of the positions of the fish were detected in the rooms, independently of the number of individuals (S1 Appendix and S16 Fig). In the Fig 2, we show that 80% of the time, less than 20% or more than 80% of the whole group is detected in the room 1. This result highlights that, as expected for a social species, the fish are not spread homogeneously in the two rooms but aggregate collectively in the patches, with only few observations of homogeneous repartition in both rooms. However, this analysis also shows that the proportion of observations with equal repartition between both rooms (40-60%) increases with the number of individuals. Thus, even if they are mainly observed together, fish in large group have a slightly higher tendency to split into subgroups. We show that the frequencies of observations for the proportions of 80 to 100% of the whole group in the room 1 are higher than 50% for all group sizes, except for 10 and 20 fish. For each trial, we defined the room 1 as the starting room where we let the fish acclimatize during 5 minutes in a transparent perspex cylinder. This may explain the observed bias of presence in favour of room 1 that may be a consequence of a longer residence time at the beginning of the trials.

**Fig 3:**
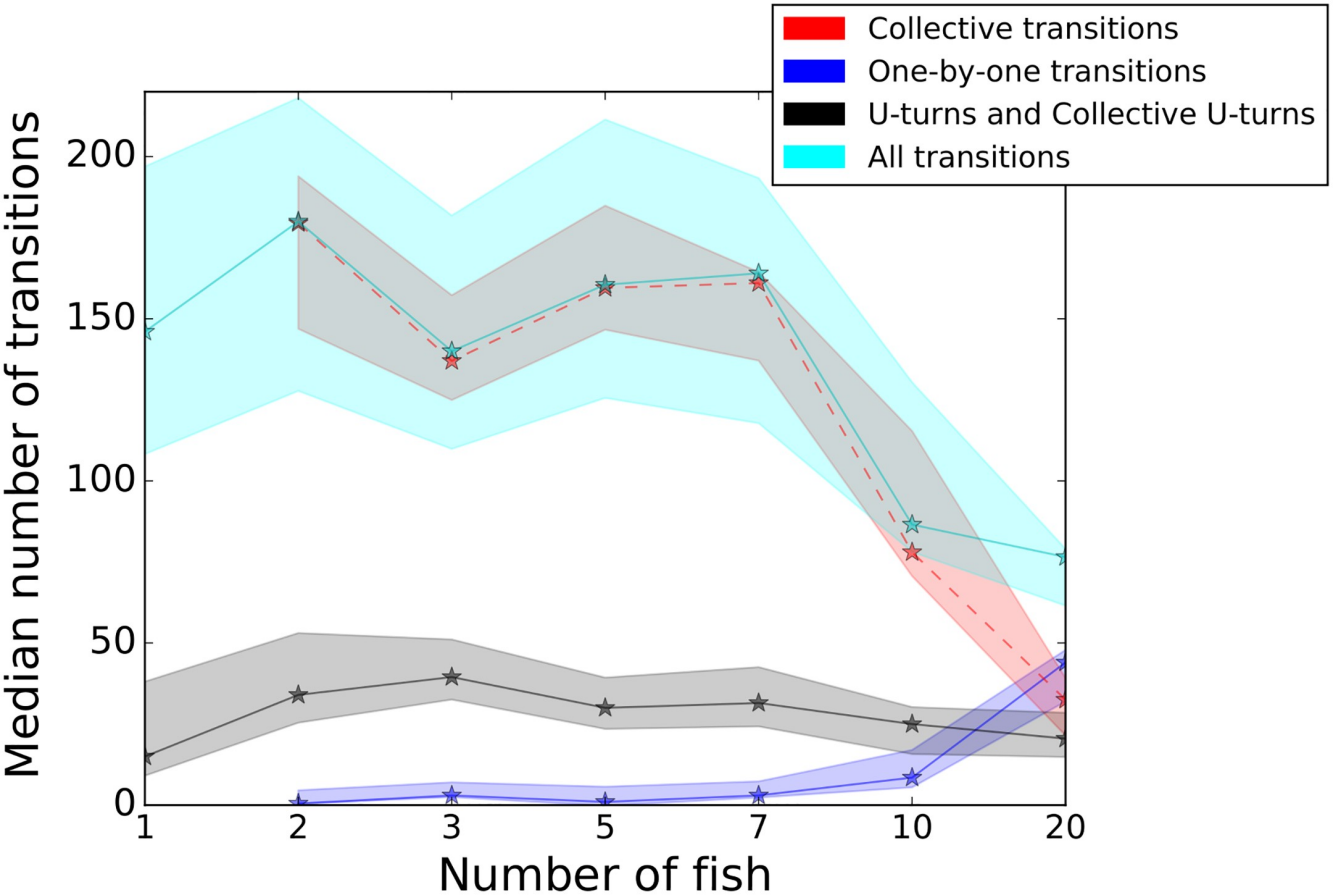
Mean and median number of transitions for groups of different numbers of individuals. The red curve shows *Collective transitions*, the blue curve shows *One-by-one transitions*, the black curve represents the *Collective U-turns* and the magenta (*All transitions*) is the sum of *Collective transitions* and *One-by-one transitions*. The stars show the medians. *One-by-one transitions* occur when the fish transit one by one from one room to the other through the corridor. *Collective transitions* appear when the whole group transit between both rooms through the corridor. *Collective U-turns* occur when the whole group go back to the previous room. The dashed lines facilitate the lecture. The figure shows that increasing the number of individuals makes the number of *Collective U-turns* and *Collective transitions* decrease and the number of *One-by-one transitions* increase. Each point shows the median of 12 values.

Then, since the fish are observed most of the time forming one group in one of the two rooms, we studied the transitions of the majority of fish between the two patches during the whole experimental time. In Fig 3, we plot the number of transitions between both rooms (see S14 and S17 Figs and S3 Table) for the plot of the means of the numbers of transitions and their standard deviations in a table). First, we present the total number of transitions (*All transitions*) for all group sizes (referred as *All transitions*). Then for groups with at least two individuals, we detailed these transitions into two subcategories: *Collective transitions* (they occur when the whole group transit between both rooms through the corridor, i.e. the majority of the group is detected successively in one room, the corridor and the other room) and *One-by-one transitions* (they occur when the fish transit one by one from one room to the other through the corridor, i.e. the majority of the group is detected successively in one room and then in the other room, indicating that the fish did not cross the corridor together). In addition, we also quantified the *Collective U-turns* that occur when the majority of the group was detected successively in one room, in the corridor and back to the previous room.

For larger groups, the numbers of *All transitions*, *Collective transitions* and *Collective U-turns* decrease while the number of *One-by-one transitions* increases. For the transitions (*Collective*, *One-by-one* and *All*), this tendency intensifies for bigger groups of 10 and 20 zebrafish. Also, for groups of 3 zebrafish, there are less *Collective transitions* (as well as *All transitions*) than for groups of 2, 5 and 7 zebrafish. *U-turns* remained rare and are very stable for all shoal sizes and their highest means are reached for groups of 2 and 3 zebrafish. *One-by-one transitions* are as well very rare for small groups and increase when the shoal size reaches 10 zebrafish.

For each number of fish (Fig 3), we compared with a Kruskal-Wallis test the distributions of the number of transitions (*Collective*, *One-by-one* and *U-turns*) and found: for 1 fish, df = 2, Chi-sq = 31.62 *p* < 0.001; for 2 fish, df = 2, Chi-sq = 30.76, *p* < 0.001; for 3 fish, df = 2, Chi-sq = 30.94, *p* < 0.001; for 5 fish, df = 2, Chi-sq = 30.41, *p* < 0.001; for 7 fish, df = 2, Chi-sq = 30.54, *p* < 0.001; for 10 fish, df = 2, Chi-sq = 19.22, *p* < 0.001 and for 20 fish, df = 2, Chi-sq = 18.36, *p* < 0.001. For each group size, we show that at least one of the distributions is significantly different from the others. The Tukey’s honest significant difference criterion shows that: all the distributions are significantly different (*p* < 0.05) except in groups of 10 individuals between *Collective U-turns* and *One-by-one transitions* and in groups of 20 individuals between *Collective U-turns* and *Collective transitions*.

**Fig 4:**
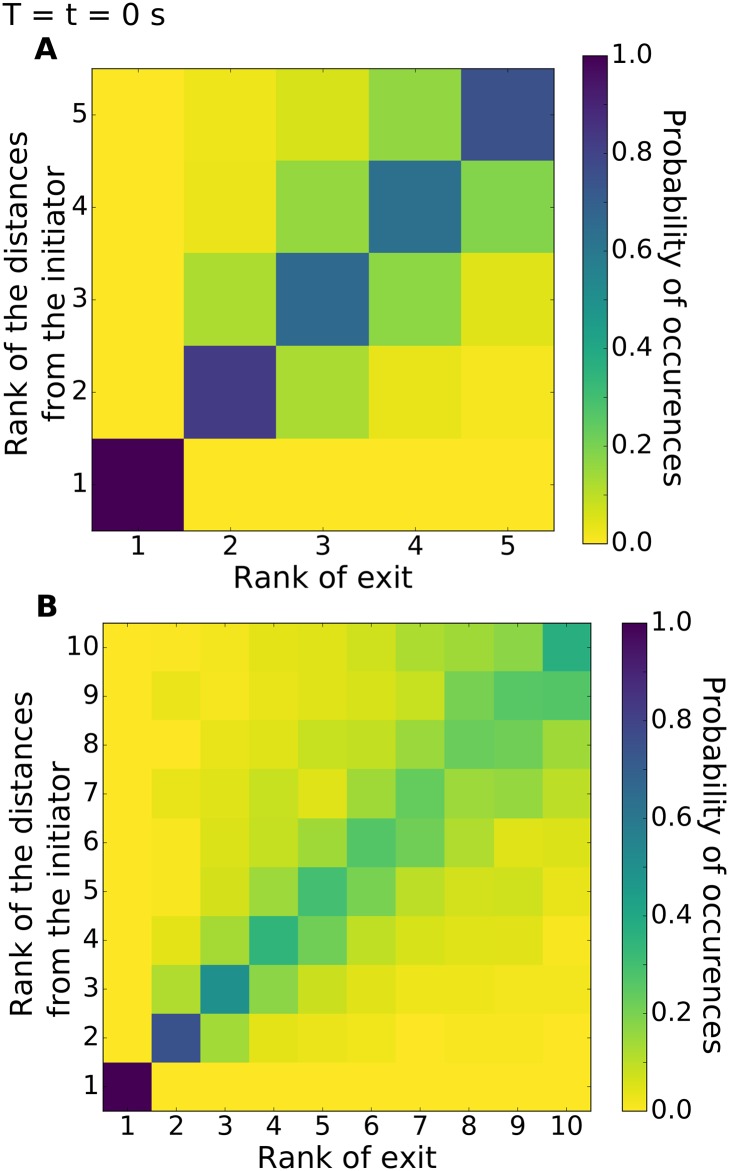
Probability of occurrence of the rank of exit with the rank of distance to the initiator for groups of 5 zebrafish (left column) and 10 zebrafish (right column). We counted N = 1456 exits for 12 replicates with 5 zebrafish and N = 277 for 12 replicates with 10 zebrafish. (A) and (B) show the map at the time when the initiator leave the room. As an example, in (A) the probability of occurrence where the second fish leaves the room and has the shortest distance from the initiator is 0.82. This probability decreases to 0.12 for fish with rank of 2 for exit and rank of 3 for distances (the second closest distance from the initiator). S18 and S19 Figs) show a more detailed version.

**Fig 5:**
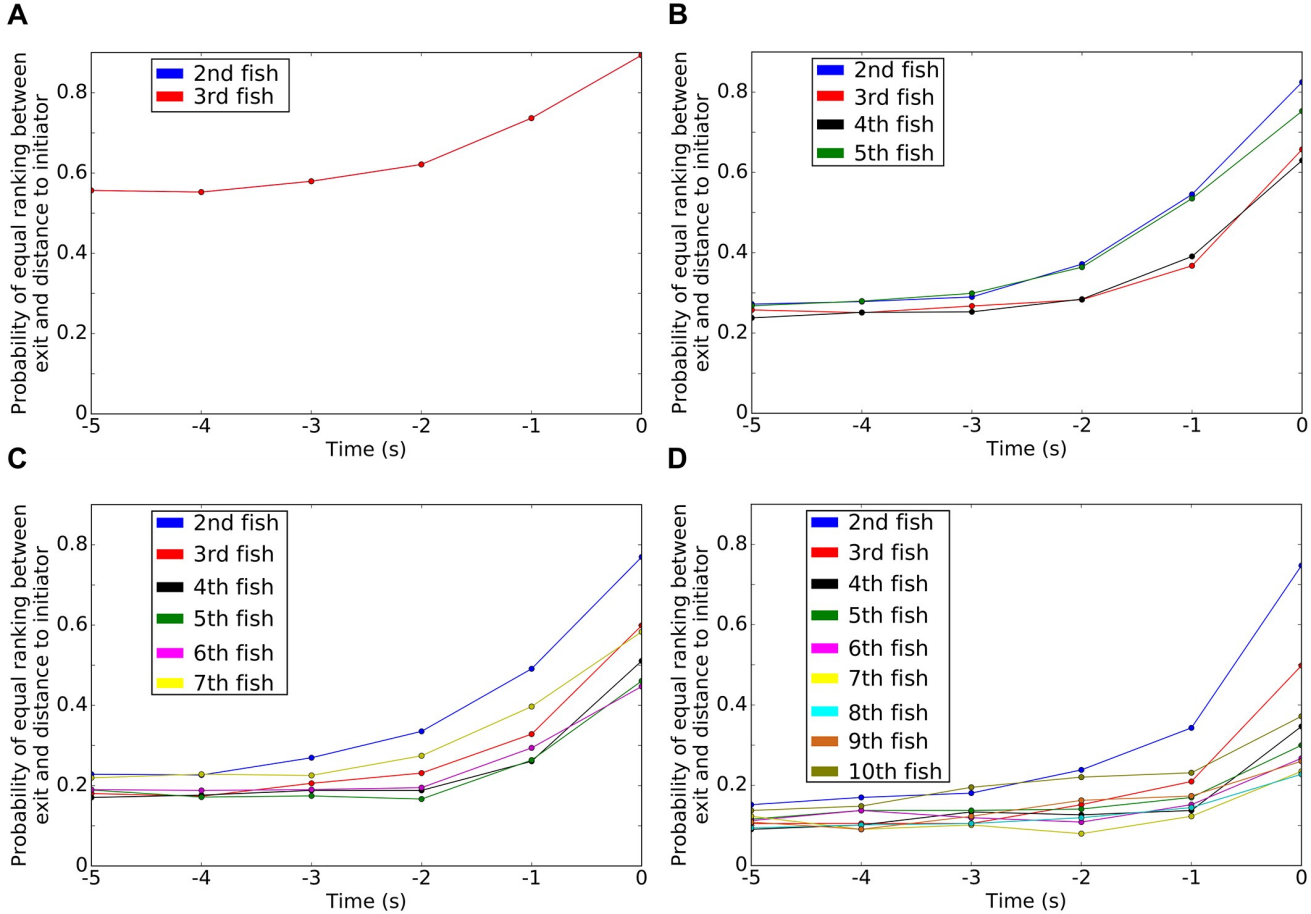
Time series of the probability of equal ranking between the rank of exit and the rank of distances from the initiator for groups of (A) 3 zebrafish, (B) 5 zebrafish, (C) 7 zebrafish and (D) 10 zebrafish. This figure is related to the results shown in Fig 4 (the diagonal). We plot a time series of the 5 seconds before the initiation. We show that the probability increases strongly 2 seconds before the initiation. We see also that this probability is the highest for the first ranked fish and higher for the 3 first ranked fish and for the last ranked fish. This behaviour is also valid even few seconds before the initiation. In (A) the line-plots for 2^*nd*^ and 3^*rd*^ fish are overlapping.

**Fig 6:**
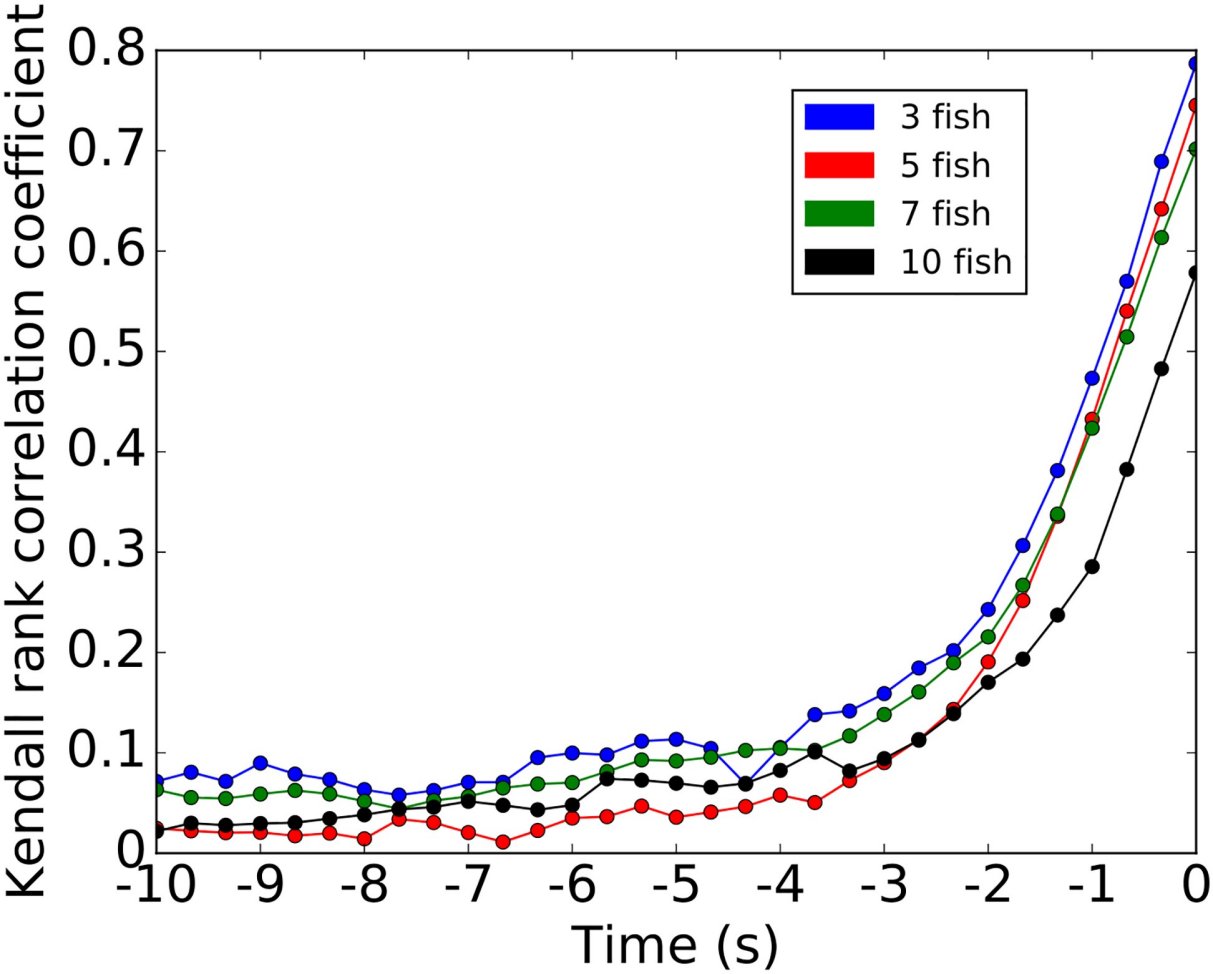
Time series of the Kendall rank correlation coefficient calculated on the results of the S18 and S19 Figs. It has been calculated every 1/3 second starting 10 seconds before the initiation. The Kendall rank correlation coefficient is a measure of ordinal association between two measured quantities. It goes to 0 when the two quantities are independent and goes to 1 if they are correlated. For example with 5 zebrafish, the time series shows that from 4 seconds before the initiation the Kendall rank increases from 0.06 to 0.75 (at T = t = 0 s). Whatever the number of individuals, we conclude that the closer to the initiation the higher the correlation between the rank of exit and the rank of the distances with the initiator.

As most of the transitions occur in groups, we analysed the dynamics of collective departures from the rooms with a particular emphasis to the pre-departure period. Thus, for each collective departure of the fish, defined as the whole group leaving one of the resting sites for the corridor towards the other one, we identified the ranks of exit of each fish and also their distances from the first fish leaving the room (i.e. defined as the initiator) measured at the departure timing of this initiator. Fig 4 represents the normalised contingency table of the rank of exit for all zebrafish from both rooms (without distinction) with the rank of the distances of all zebrafish to the initiator. These results correspond to 12 replicates of groups of 5 and 10 zebrafish. The initiator has a rank of exit and a rank of distances of 1. For example, in (A) the probability that the first fish to follow the initiator (rank 2) was also the closest fish of the initiator when it exited the room is 0.82. As evidenced by the darker diagonal of the contingency matrix, the rank of exit was closely related to the distance from the initiator at the beginning of the departure. S18 and S19 Figs) show a more detailed version of the Fig 4 for 3, 5, 7 and 10 individuals. In Fig 5, we plot for 3, 5, 7 and 10 zebrafish the values of the probability of equal ranking between the exit and the distances with the initiator (i.e. the diagonal of the previous plots—Fig 4) for different time-lag before the exit of the initiator. In particular, we computed the ranks of the distances from the initiator at 1 to 5 seconds before the exit of the initiator. First, these measures show that the further from the time of the initiation the lower the probability of equal ranking. This assessment is valid for every shoal sizes. Second, we see that the probability of equal ranking is often higher for the first and for the last ranked fish even few seconds before the initiation (around 2 seconds before the initiation). In Fig 6, we use the Kendall rank correlation coefficient to see if the rank of exit and the rank of distances with the initiator are dependent (close to 1) or not (close to 0) through the time. For every group sizes, we show an increase of the Kendall rank correlation coefficient when closer to the initiation. For 3 zebrafish, the time series show that from 4 seconds before the initiation the Kendall rank correlation coefficient fully increases from 0.11 to 0.79 (at T = t = 0 s). For 5 zebrafish, it increases from 0.06 (at T = t − 4 s) to 0.75 (at T = t = 0 s), for 7 zebrafish, it increases from 0.10 to 0.70 and for 10 zebrafish, it increases from 0.08 to 0.58. These results show that for all group sizes, the closer to the initiation the higher the correlation between the rank of exit and the rank of the distances with the initiator.

## Discussion

We studied the impact of the number of individuals in the shoals (1, 2, 3, 5, 7, 10 or 20 individuals) on the collective motion and the collective departure between two environmental patches in adult AB zebrafish.

Here we consider group size as the number of fish in a group. By changing that number of fish it may lead to size or density effects. We do not adress the question of untangling size or density effects in this study. The density is related to a need of individual space and the group size is related to a limitation in the considerations, for each individual, of the other members of the group. Our set-up reached a compromise between reducing the size of the set-up for small numbers of individuals, that would have an effect on the small group collective transitions, and increasing the size of the set-up, that would have led to tracking issues (less pixels by fish) [49]. Actually, if we resize the set-up proportionally to the density, we will modify the lengths and the widths of the rooms and the corridor. This would have an effect on the time of residency in the rooms or on the decision to cross the corridor and would introduce new variables in the experiment.

Furthermore, the Fig 1, showing that the more individuals the lower the nearest neighbour distances, elucidates the previous statement. For small groups, the medians of the nearest neighbour distances distributions decrease when increasing the numbers of individuals. This result could be due to an effect of the set-up where the environment is not resized in proportion of the number of the individuals. For groups of at least 5 fish, it seems that the medians of the nearest neighbour distances distributions are very similar. Here, it is possible that the threshold value of the nearest neighbour distance for the AB zebrafish has been evidenced. In this latter case, it means that the zebrafish spread more in the set-up which show that the groups are not denser when we increase their sizes. Moreover, the results of the Two-Way ANOVA evidenced that the influence of the number of individuals over the nearest neighbour distances was significant when the influence of the type of the area (rooms or corridor) is not. In [50], the authors have shown that the mean of the nearest neighbor distances is about 30 mm for groups of 8 wild type zebrafish (presumably AB). Their results are very close from ours and the experiments were performed in a circular shape bowl of 20 cm in diameter. Both of our results may show that the environment has no influence on the compact structure of big groups. In parallel, if we focus on the distances between all pairs within a group we show that the higher the number of individuals the higher the medians of the distances between all respective pairs of zebrafish (S4, S5, S6, S7, S8 and S9 Figs). Finally, we show that the higher the number of individuals the lower the time the fish stay with their nearest neighbours (S10 Fig). The combination of these results shows that there is a clear effect of the number of individuals on the cohesion; an effect that we already have shown in [37] where the bigger the group the higher the cohesion of the whole group.

It seems that there are preferential interactions between zebrafish (S4, S5 and S6 Figs) and increasing the number of individuals will affect these interactions: respective pairs are less cohesive in larger groups. We considere that revealing the time spent by the fish with their nearest neighbours show stronger informations about preferences between pairs than the median distances of each couple, which are already approximations. Hence, we show (S10 Fig) that for goups of 3 fish the distribution of the number of times the fish stay with their nearest neighbours is not significantly different from a random uniform distribution. It means that for such number of individuals there is no preferential interaction. For bigger groups, these distributions are significantly different from a random uniform distribution which means that there are preferential interactions. Preferential interactions have been evidenced in other species: Briard et al. [51] show affinities, hierarchy and pairs interactions in a group of domestic horses, [52–55] show that the affinity between individuals (monkeys, *Macaca mulatta*, *Macaca tonkeana*, *Papio ursinus*; or fish, *Gasterosteus aculeatus*) play a role in the collective movements. We propose two hypotheses that could explain the change of the interactions between pairs of zebrafish when changing the number of individuals. On the one hand, in groups larger than two fish, each zebrafish has to choose the preferred partners, between all other fish. In larger groups there are more individual choices and more preference tests. On the other hand, the patchy environment may break pair interactions and may force the emergence of new pairs. These two hypotheses could explain the dynamics of the pair interactions observed during the experiments.

The fish are detected 70% of the time in the rooms (S16 Fig). On average, they spend about 10 seconds in a room (S15 Fig), transit to the other room through the corridor (4 seconds on average) and then come back. They oscillate between the rooms. In a previous study we showed that zebrafish also transit and oscillate between landmarks in an open environment [37]. The Fig 3 shows that most of the transitions are collective when the Fig 2 shows that the whole group swim generally together in both rooms. This observation is strengthened by the very rare number of *One-by-one* transitions between the rooms. However, groups of 10 and 20 zebrafish show sharp decreases in the number of collective transitions. This drop could be due to the topology of the set-up and congestion effects. Larger groups can split into smaller subgroups. The threshold we imposed in the analysis of the collective transitions (below 70% of the whole group, the transitions were not taken into account) may reinforce this effect. This seems to be confirmed by the S17 Fig which shows the mean and median number of transitions for different numbers of individuals when all the fish start to move from a room: the larger the group, the lower the number of transitions with the whole group. Also the Fig 2 shows that the bigger the groups, the higher the frequency of observations of sub-groups located in the rooms and in the corridor (20-40%, 40-60% and 60-80%). Many studies have analysed the fusion-fission mechanisms occurring in groups of fish or mammalians. In [56–58], the authors show that these mechanisms are frequent in the wild and generate body length assortment within groups of fish (*Fundulus diaphanus*, *Notemigonus crysoleucas*, *Catostomus commersonii*, *Poecilia reticulata*). Sueur et al. [8] show that fission-fusion mechanisms participate in the information transfer between subgroups and the group of *Myotis bechsteinii*. In the corridor, we observe few U-turns. The zebrafish swimming preferentially along the walls and a canalisation effect of the corridor may explain this observation. As expected, connecting the two patches, the corridor is used as a mere transit area (zebrafish show higher speeds in the corridor—S1, S2, S3 and S12 Figs).

We show that the organisation of the group during collective departures takes place during a short pre-departure period and is related to the distances between the initiator (of the exit from the room) and the other fish. Ward *et al*. have shown that the first fish (of a group of 5 *Dascyllus aruanus*) to follow the initiator is generally (rank = 2: 53% of the trials over 2 trials for each 15 groups of fish) the nearest neighbour of the initiator and that the frequency of equality between the rank of exit and the rank of the distances from the initiator decreases, with these results rank = 3: 27% and 33%, rank = 4: 20% and 7% then rank = 5: 0% and 7%) [26]. We tested four different numbers of individuals (3, 5, 7 and 10 zebrafish) and show similar results especially on the decreasing trend of the probability when focusing on the next ranked fish (S18 and S19 Figs) and Fig 5). However, we observed an extremely high probability of equal ranking for the first fish that follows the initiator (rank = 2: 75% to 90%), high probabilities of equal ranking for the second and the last fish that follow the initiator (rank = 3: 50% to 65% and rank = last fish: 38% to 75%) and showed that the probabilities of equal ranking for the other fish are quite similar to each others. The rank of exit and the rank of the distances from the initiator are strongly correlated at the moment of the exit (T = 0s, Fig 6), from 60% to 80%. Hence, it seems that the organisation of the zebrafish groups (2^*nd*^, 3^*rd*^ and last ranked fish) during the collective departures is topological. Other studies about the organisations of collective departures show a joining process for *Equus ferus caballus* that is related to affinities and hierarchical ranks [51]. Rosenthal *et al*. show that, in groups of *Notemigonus crysoleucas*, the initiator is the closest fish from the group boundary in 27% of the cases and the first responder is the closest fish from the group boundary in 19% of the cases [29]. Moreover during the initiation, when fish leave the rooms, our results suggest the idea of cascades of behavioural changes already developed by Rosenthal *et al*. [29]: the initiator drags another fish along that drags another one, etc.

This organisation appears a few seconds before the fish leave a room to transit to the other one. Two seconds before the initiation, the groups show a structure that prepares for the exit (Fig 5). The Kendall rank correlation coefficient confirmed the idea of the organisation as it reaches 18% to 25% two seconds before the departure and 30% to 50% one second before the departure (Fig 6). In the literature we found other cases of initiations: [20, 59] have shown that a three-spined stickleback *Gasterosteus aculeatus* or a sheep *Ovis aries* alone moving away from the herd can initiate a collective departure, [60] have noticed a large variety of initiations for groups of mountain baboons *Papio ursinus* where the initiator can be joined by the group immediately or [61, 62] have observed for white-headed capuchins *Cebus capucinus* a synchronization of their behaviours and also that a minimum proportion of the whole group is needed to launch a collective departure.

In conclusion, this study showed that the number of fish affects the motion of each individual in the groups and the group cohesion. The analysis of the dynamics showed that the zebrafish oscillate mainly in groups between the two patches in the environment and that the majority of the departures is collective. During the collective departures, we observed that an intra-group organisation appears prior to the transition. Increasing the number of individuals makes this organisation less predictable. Finally, we noticed that a few seconds before the collective departures the groups have a particular spatial organisation.

## Methods

### Fish and housing

We bred 600 AB strain laboratory wild-type zebrafish (*Danio rerio*) up to the adult stage and raised them under the same conditions in tanks of 3.5L by groups of 20 fish in a zebrafish aquatic housing system (ZebTEC rack from Tecniplast) that controls the water quality. It changes 10% of the water in the breeding tanks every hour. Zebrafish descended from AB zebrafish from different research institutes in Paris (Institut Curie and Institut du Cerveau et de la Moelle Épinière). AB zebrafish show zebra skin patterns and have short tail and fins. They measured in mean = 3.0 cm ± 0.36 cm, median = 2.9 cm long. All zebrafish used during the experiments were adults from 7 to 8 months of age. We kept fish under laboratory conditions: 27°C, 500*μ*S salinity with a 10:14 day:night light cycle, pH is maintained at 7.5 and nitrites (NO^2−^) are below 0.3 mg/L. Zebrafish are fed two times a day (Special Diets Services SDS-400 Scientic Fish Food).

### Experimental setup

**Fig 7:**
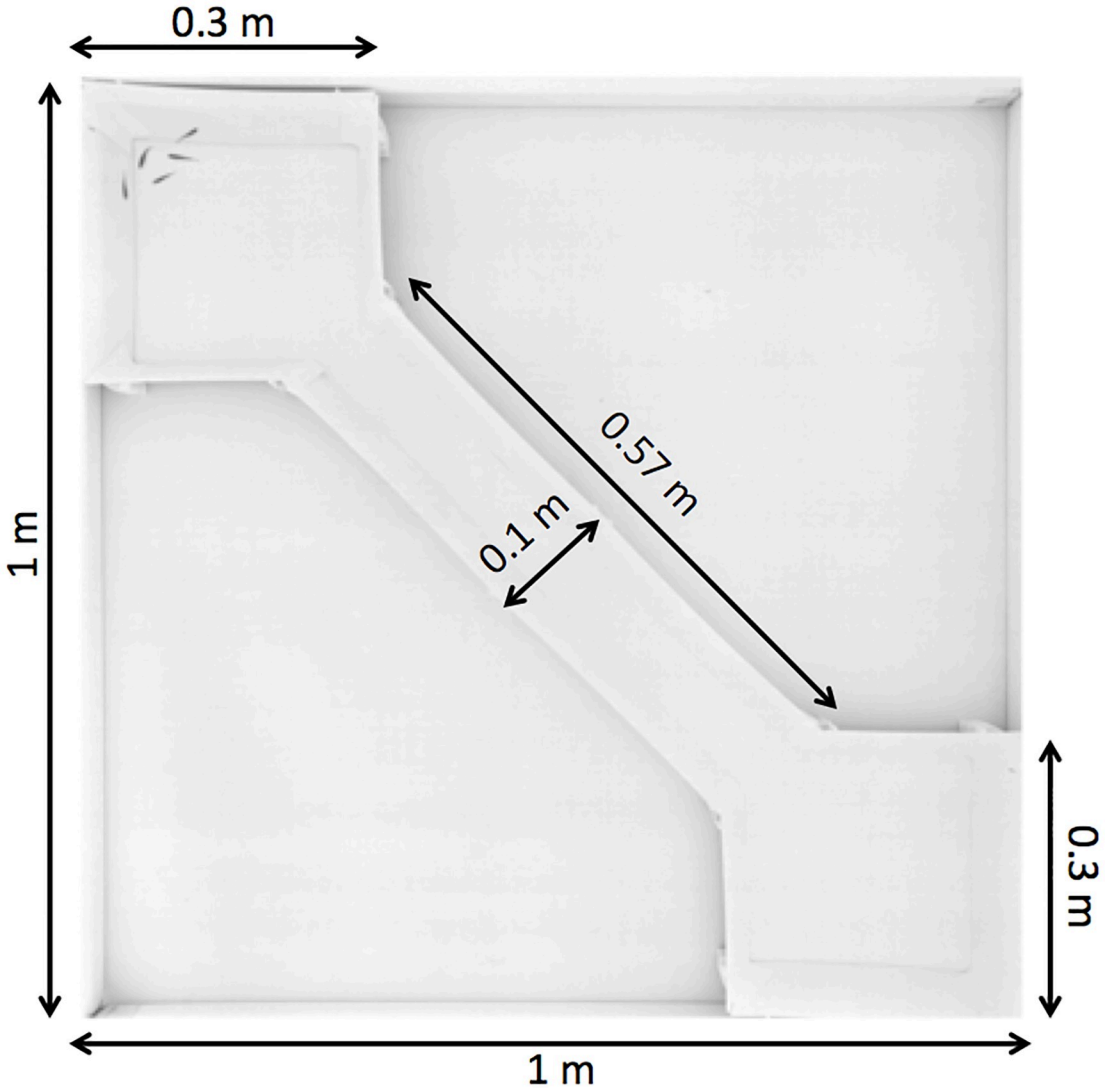
Experimental setup. A tank of 1 m x 1 m is divided into three areas: two rooms (0.3 m x 0.3 m) connected by a corridor (0.57 m x 0.1 m). The water column has a height of 6 cm. The luminosity is ensured by 4 LED lamps of 33*W* (LP-500U) placed on corners of the tank and directed towards the walls to provide indirect lighting. The whole setup is confined in a 2 m x 2 m x 2.35 m experimental chamber surrounded by white sheets to isolate the experiments and to homogenise luminosity.

The experimental tank consisted in a 1.2 m x 1.2 m tank confined in a 2 m x 2 m x 2.35 m experimental area surrounded by white sheets, in order to isolate the experiments and homogenise luminosity. A white opaque perspex frame (1 m x 1 m x 0.15 m—interior measures) is placed in the center of the tank. This frame helped us to position the two rooms and the corridor. The squared rooms (0.3 m x 0.3 m) and the corridor (0.57 m x 0.1 m) have been designed on Computer-Aided Design (CAD) software and cut out from Poly(methyl methacrylate) (PMMA) plates of 0.003 m thickness. Each wall are titled, (20° from the vertical) to the outside with a vertical height of 0.14 m, to avoid the presence of blind zones for the camera placed at the vertical of the tank (Fig 7). The water column had a height of 6 cm, the water pH is maintained at 7.5 and Nitrites (NO^2−^) are below 0.3 mg/L. The experiments are recorded by a high resolution camera (2048 px x 2048 px, Basler Scout acA2040-25gm) placed above the experimental tank and recording at 15 fps (frame per second). The luminosity is ensured by 4 LED lamps of 33W (LED LP-500U, colour temperature: 5500 K–6000 K) placed on each corner of the tank, above the aquarium and directed towards the walls to provide indirect lightning.

### Experimental procedure

We recorded the behaviour of zebrafish swimming in the setup during one hour and did 12 replicates with groups of 1, 2, 3, 5, 7, 10 and 20 zebrafish. Each fish is tested only once. Every six replicates out of twelve we rotated the setup by 90° to avoid a potential bias associated with the initial position of the tank or environmental bias (noise, light, vibrations, …). For each replicate we choose randomly the starting chamber by flipping a coin. We called the starting chamber *Room 1*. Heads or tails defined the starting chamber with a maximum of three starts in each chamber. This method gave us 3 experiments with each combination of orientation of the setup x starting chamber. Then, the fish are placed with a hand net in a cylindrical arena (20 cm diameter) made of Plexiglas in the centre the selected rooms. Following a five minutes acclimatisation period, this cylinder is removed and the fish are free to swim in the setup. The fish are randomly selected regardless of their sex and each fish is never tested twice to prevent any form of learning. The water of the tank is changed every week and the tank and the set-up are cleaned during the process.

### Tracking & data analysis

Today, many studies on animal collective behaviours use methodologies based on massive data gathering, for exemple with flies (*Drosophila melanogaster*) [63, 64], birds (*Sturnus vulgaris*) [65–67], fish (*Notemigonus crysoleucas*) [68]. Our experiments are tracked in real-time (“on-line”) by a custom made tracking system based on blob detection. Each replicate except experiments with 20 zebrafish is also tracked by post-processing (“off-line”) with the idTracker software to identify each fish and their positions [49]. Each replicate consisted of 54000 positions (for one zebrafish) to 1080000 positions (for 20 zebrafish). The idTracker software is not used for groups of 20 fish due to higher number of errors and too long computing time. For example, for a one hour video with 2 fish idTracker gives the results after 6 hours of processing and for a one hour video with 10 fish it lasts a week to do the tracking (with a Dell Precision T5600, Processor: Two Intel Xeon Processor E5-2630 (Six Core, 2.30GHz Turbo, 15MB, 7.2 GT/s), Memory: 32GB (4x8GB) 1600MHz DDR3 ECC RDIMM).

Since idTracker solved collisions with accuracy [49] we calculated individual measures and characterised the aggregation level of the groups (except for groups of 20 individuals). We also calculated the distances between each pair of zebrafish respectively, the travelled distances (S11 Fig) of each individual and their speeds (S1, S2, S3 and S12 Figs). The computing of the speed has been done with a step of a third of a second in sort of preventing the bias due to the tracking efficiency of idTracker that does not reach 100% (see S2 Table). The data gathered for groups of 20 individuals are only used in the analyses which focused on group behaviour and do not need the identities of the fish. The data analysts were not blind to group size. However, the data analysis was done automatically by custom scripts that did not differentiate between the different experimental conditions. Each .txt with the position (x,y,t) of the fish entered the pipeline analysis and received the same treatment. Thanks to this automated treatment, we ensured that there was no bias in the data analysis due to the observer.

The Fig 2 has been obtained following this process: At each time step with at least one fish detected in a room, we analysed the repartition of the group among the rooms by computing the proportion of fish present in room 1 = *R*_1_ / (*R*_1_+*R*_2_) with *R*_1_ and *R*_2_ the number of fish in the respective room number.

When all fish were present in the same room, we identified which fish initiates the exit from the room, established a rank of exit for all the fish and calculated the distances between all zebrafish to the initiator to establish a rank of distances. Finally, we confronted these ranks and count the number of occurences for each ranking case. We checked the results for different time steps before the initiation. The idea was to highlight a correlation between the spatial sorting and the ranks of exit and also a possible prediction of ranks of exit.

We looked at majority events defined as the presence of more than 70% of the zebrafish in one of the three areas of the setup, either in the room 1 or in the room 2 or in the corridor. To compute their numbers, we averaged the number of fish over the 15 frames of every second. This operation ensures that a majority event is ended by the departure of a fish and not by an error of detection during one frame by the tracking system. We then computed the durations of each of those events and counted the transitions from a room to the other one and sort them. All scripts were coded in Python using scientific and statistic libraries (numpy, pylab, scilab and matplotlib).

Finally, we computed the neighbour distances as the distances between each nearest fish (S13 Fig).

### Statistics

All scripts were coded in Matlab and Python using statistics libraries (numpy, pylab, scilab and matplotlib). For the S12 Fig we report the number of values of the speeds on the S1 Table. For the Figs 1 and 3 we tested the distributions using Kruskal-Wallis tests completed by a post-hoc test: Tukey’s honest significant difference criterion. For the S12 Fig we tested with ANOVA N-way 10 samples of 0.5% of the data of the speed choosen randomly for each number of individuals.

The Kendall rank correlation coefficient [69], *τ*, is a measure of ordinal association between two measured quantities. It goes to 0 when the two quantities are independent and to 1 if they are correlated. It is computed by:

$$\tau =\frac{\text{number}\;\text{of}\;\text{concordant}\;\text{pairs}\;-\;\text{number}\;\text{of}\;\text{discordant}\;\text{pairs}}{\text{number}\;\text{of}\;\text{concordant}\;\text{pairs}\;+\;\text{number}\;\text{of}\;\text{discordant}\;\text{pairs}}.$$

We use the Kendall rank correlation coefficient to see if the rank of exit and the rank of distances with the initiator are dependant or not through the time.

## Animal ethics

The experiments performed in this study were conducted under the authorisation of the Buffon Ethical Committee (registered to the French National Ethical Committee for Animal Experiments #40) after submission to the state ethical board for animal experiments.

## Supporting information

S1 Appendix Appendix of the main article with the 19 figures ans 3 tables listed below. (PDF)

S1 Fig Distribution of the individual speeds for an individual and a pair of AB zebrafish. (TIFF)

S2 Fig Distribution of the individual speeds for two group sizes. Groups of 3 and 5 AB zebrafish.
(TIFF)

S3 Fig Distribution of the individual speeds for two group sizes. Groups of 7 and 10 AB zebrafish.
(TIFF)

S4 Fig Medians of the distances between all respective pairs of zebrafish per trial in the room 1 for 5 different group sizes. (TIFF)

S5 Fig Medians of the distances between all respective pairs of zebrafish per trial in the room 2 for 5 different group sizes. (TIFF)

S6 Fig Medians of the distances between all respective pairs of zebrafish per trial in the corridor for 5 different group sizes. (TIFF)

S7 Fig Medians of the distances between all respective pairs of zebrafish per trial in the room 1 for 5 different group sizes. (TIFF)

S8 Fig Boxplots of the medians of the distances between all respective pairs of zebrafish per trial in the room 2 for 5 different group sizes. (TIFF)

S9 Fig Boxplots of the medians of the distances between all respective pairs of zebrafish per trial in the corridor for 5 different group sizes. (TIFF)

S10 Fig Boxplots of the number of times the fish stay with their nearest neighbours for 4 different group sizes. (TIFF)

S11 Fig Mean and median cumulative travelled distances for different group sizes. (TIFF)

S12 Fig Mean and median of the individual speeds for different group sizes. (TIFF)

S13 Fig Distributions of the nearest neighbour distances. (TIFF)

S14 Fig Means of majority events with a majority of zebrafish in the three areas, for 7 group sizes. (TIFF)

S15 Fig Means of the time spent by a majority of fish in each area. (TIFF)

S16 Fig Proportion of fish detected in both rooms. (TIFF)

S17 Fig Mean and median number of transitions for different group sizes. (TIFF)

S18 Fig Probability of occurrence of the rank of exit with the rank of distances from the initiator. (TIFF)

S19 Fig Probability of occurrence of the rank of exit with the rank of distances from the initiator. (TIFF)

S1 Table Number of values of speeds. (TIFF)

S2 Table Mean tracking efficiency. (TIFF)

S3 Table Standard deviations of the means of majority events, durations, numbers of transition types with a majority of zebrafish in the three sections of the setup. (TIFF)
