# Supplementary material for: Loose social organisation of AB strain zebrafish groups in a two-patch environment
Source: PLoS One. 2019 Feb 8;14(2):e0206193. doi: 10.1371/journal.pone.0206193 (PMC6368274; doi:10.1371/journal.pone.0206193)
Supplement: S1 Appendix — (PDF) [file pone.0206193.s001.pdf]

I. Supporting Information

Supplementary figures of "Loose social organisation of AB strain zebrafish groups in a two patches environment".

A. Group structure and group size

FIG. 1: **Distribution of the individual speeds for an individual and a pair of AB zebrafish.**

FIG. 2: **Distribution of the individual speeds for two group sizes.** Groups of 3 and 5 AB zebrafish.

FIG. 3: **Distribution of the individual speeds for two group sizes.** Groups of 7 and 10 AB zebrafish.

FIG. 4: **Medians of the distances between all respective pairs of zebrafish per trial in the room 1 for 5 different group sizes.** Each square corresponds to the median of the distances between the fish of one specific pair during a whole trial (out of 12 trials). When the colour of the square is yellow it means that the median of the distances between the fish is small (0.05m), when the colour is dark-blue it means that the median of the distances is larger (0.15m). For each area, we see a large distribution of the medians of the distances and their increase with the group size. As the tracking software is able to individually recognise the different group members during the entire experiment, we were able to measure the distance between each specific pair of fish from the beginning to the end of the observation period. These distances are presented in S4, S5 and S6 Figs of the appendix.

FIG. 5: **Medians of the distances between all respective pairs of zebrafish per trial in the room 2 for 5 different group sizes.**

FIG. 6: **Medians of the distances between all respective pairs of zebrafish per trial in the corridor for 5 different group sizes.**

FIG. 7: **Medians of the distances between all respective pairs of zebrafish per trial in the room 1 for 5 different group sizes.** We compared with a Kruskal-Wallis test the distributions and found: df = 4, Chi-sq = 486.47 and $p < 0.001$. We show that the distribution of the medians of the distances between pairs of fish are not significantly different for groups of 2, 3 and 5 fish and significantly different for groups of 7, 10 and 2, 3 or 5 fish.

FIG. 8: **Boxplots of the medians of the distances between all respective pairs of zebrafish per trial in the room 2 for 5 different group sizes.** We compared with a Kruskal-Wallis test the distributions and found: df = 4, Chi-sq = 500.73 and $p < 0.001$. We show that the distribution of the medians of the distances between pairs of fish are not significantly different for groups of 2, 3 and 5 fish and significantly different for groups of 7, 10 and 2, 3 or 5 fish.

FIG. 9: **Boxplots of the medians of the distances between all respective pairs of zebrafish per trial in the corridor for 5 different group sizes.** We compared with a Kruskal-Wallis test the distributions and found: df = 4, Chi-sq = 511.29 and $p < 0.001$. We show that the distribution of the medians of the distances between pairs of fish are not significantly different for groups of 2, 3 and 5 fish and significantly different for groups of 7, 10 and 2, 3 or 5 fish.

FIG. 10: **Boxplots of the number of times the fish stay with their nearest neighbours for 4 different group sizes.** For the groups of 10 fish, there are rwo outliers. In two different trials, one pair of zebrafish spent 46910 (52 minutes) and 15050 (17 minutes) frames together. We compared with a Kruskal-Wallis test the distributions and found: df = 3, Chi-sq = 659.42 and $p < 0.001$. We show that the distribution of the number of times the fish stay with their nearest neighbours are not significantly different for groups of 3 and 5 fish and significantly different for groups of 7, 10 and 3 or 5 fish. We compared with the Wilcoxon rank sum test the 36 measured values of the time spent by each couple for groups of 3 fish with 36 random values from a uniform distribution included between the minimum and the maximum of the measured values. We show that zval = -1.2895, ranksum = 1199 and $p > 0.1$, which means that both of the distributions are not significantly different. With the same methodology, we compared the 120 mesured values of the trials with 5 fish with 120 values from a uniform distribution and found: zval = -4.3225, ranksum = 12135 and $p < 0.001$, which means that both of the distributions are significantly different. We compared the 252 mesured values of the trials with 7 fish with 252 values from a uniform distribution and found: zval = -8.7288, ranksum = 49360 and $p < 0.001$, which means that both of the distributions are significantly different. We compared the 450 mesured values of the trials with 10 fish (after removing the two outlier trials) with 450 values from a uniform distribution and found: zval = -13.8971, ranksum = 148536 and $p < 0.001$, which means that both of the distributions are significantly different.

We showed that the distances travelled by the zebrafish are related to the size of the group (S11 Fig of the appendix). Groups of 2 to 7 zebrafish travelled the longer distances (with a declining trend) and fish alone and groups of 10 zebrafish travelled the shorter distances.

FIG. 11: **Mean and median cumulative travelled distances for different group sizes.** The blue points represent the means, the red dot the medians. Small groups of zebrafish travel more than bigger groups and fish alone. Error bars show the standard deviation.

S12 Fig shows the means and the medians of the individual speeds of the fish measured during the entire experimental time (one hour) and according to their spatial location (in the corridor or in one of the two rooms). The fastest individuals are observed in groups of 5 fish in the corridor and 3 fish in both rooms. On the contrary, fish alone and groups of 10 individuals show the slowest mean and median speeds. Moreover in the corridor, between the group sizes of 1 and 5 individuals, there is an increase of the mean and median speed. Then, for bigger group sizes, the means and medians decrease. Likewise, in both rooms, for the group sizes of 1 and 3, we observe an increase of the mean and the median of the speeds, then for bigger group sizes a drop.

We tested with ANOVA N-way (which is a generalisation of the ANOVA Two-way and because of the unbalanced size of the samples) 10 samples of 0.5% of the data of the speed randomly choosen for each group size. For the location (room 1, room 2 and corridor), df = 2, the F-value oscillates between 272.98 and 350.67, for the group size, df = 5, the F-value oscillates between 45.86 and 90.46, for the interaction, df = 10 and F-value oscillates between 2.07 and 4.56. There is an effect of the size of the groups and another one of the area (rooms or corridor) on the speed (p-value < 0.005). There is a small effect of the interaction of the size of the groups and the area on the speed (p-value < 0.02).

We show that the individual speed of the zebrafish varies according to the areas in which they are swimming and their group size. The zebrafish move faster in the corridor and have similar lower speeds in both rooms (S12 Fig of the appendix). The surface of the corridor is the third of a room and it constraints the direction the fish have to follow. We have shown that zebrafish are known to swim along the walls of the experimental tank [1, 2] thus showing a strong thigmotaxis. In the corridor, that can be compared to a tunnel, canalised by the walls of the corridor, the zebrafish increase their individual speeds to make the transit from one room to the other. In both rooms the means and the medians of the individual speeds are at their highest levels for groups of 3 zebrafish and the maximum of the means and the medians of the individual speeds is reached for group size of 5 fish in the corridor. In parallel, in each area these means and medians are at their lowest levels for the smallest and the biggest group sizes: 1 and 10 zebrafish. Hence, in both rooms and in the corridor respectively, we have seen that from 1 to 3 individuals and from 1 to 5 individuals the individual speeds increase, when from 3 to 10 individuals and from 5 to 10 individuals, the individual speeds decrease.

First, our results confirm that the behaviour of a zebrafish alone differs significantly from the behaviour of zebrafish in groups. Isolated zebrafish travel a shorter distance and at a lower speed than zebrafish in groups. This can be the result of the stress generated by being isolated in a new environment. The stress level has been studied and [3] shows that some anxiolytics (fluoxetine and ethanol, which reduce stress level) will increase the speed and/or the travelled distances of zebrafish alone in a tank. Second, zebrafish swim faster in smaller group sizes and their speeds decrease for bigger group sizes. We observe the same trend for the travelled distances. These results may suggest a congestion effect where obstruction can affect their individual speeds and hence their travelled distances during the experimentation time [4]. Such effect has already been reported for example in the ant species *Atta cephalotes*: crowded conditions on the trail network make the velocity decrease [5]. Herbert-Read et al. present another explanation for the changes of the motions where each fish (*Gambusia holbrooki*) conforms to the group behaviour

through the interaction rules between the individuals and the decisions of each individual to follow or copy their neighbour movements [6]. Although this case seems to be extreme, [7–9] have shown that fish from different species (*Perca fluviatilis*, *Gasterosteus aculeatus*) and *Gambusia holbrooki* can maintain particular individual behavioural traits in a social context. These changes in behaviours are found in other animal species such as birds (*Erythrura gouldiae*) that adjust their behaviour according to the personality of their partners [10].

FIG. 12: **Mean and median of the individual speeds for different group sizes.** The red dots represent the mean and the red stars the medians of the individual speed for all individuals in the corridor, the blue dots represent the mean and the blue stars the medians for the room 1 and the black dots represent the mean and the black stars the medians line for the room 2. The zebrafish move faster in the corridor. In rooms 1 and 2, their speeds are similar. Groups of 3 zebrafish show the highest speeds in both rooms and groups of 5 zebrafish the highest speeds in the corridor.

TABLE 1: **Number of values of speeds**. This table is related to S12 Fig of the article.

FIG. 13: **Distributions of the nearest neighbour distances.** Groups of (A) 2 AB zebrafish, (B) 3 AB zebrafish, (C) 5 AB zebrafish, (D) 7 AB zebrafish, (E) 10 AB zebrafish and (F) 20 AB zebrafish. The plots are based on 648012 distances for 12 replicates. The dashed lines represent the medians. The distributions show for groups of 5, 7, 10 and 20 zebrafish similar medians and a shift to higher median values for smaller groups: 2 and 3 zebrafish. The nearest neighbour distances refer to the shortest distances between all zebrafish at every time step. It is a measure of group cohesion. The interest of such analysis is to dismiss the effect of the geometry of the set-up and to focus only on the group bearing.

B. Oscillations and collective departures

To analyse the dynamics of the space occupancy in the set-up we computed the mean number of majority events and the mean durations and cumulative durations of occupancy by a majority of individuals within the three areas when a majority of the whole group is reached (S14 and S15 Figs). We define the majority as 70% of the individuals being present in the considered section of the set-up. On the S14 Fig, we find more majority events in the corridor than in room 1 or room 2 except with groups of 20 zebrafish. Whatever the size of the group, we find almost the same number of majority events inside the rooms 1 and 2. Also, in all areas we see that for groups of 10 and 20 zebrafish the bigger the group the lower the number of majority events. The difference between the number of majority events in the corridor and in both rooms is relatively stable for groups of 2, 3, 5 and 7 zebrafish but decreases when increasing the size of the groups (10 to 20 zebrafish). The mean number of majority events finally reaches almost the same value when 20 zebrafish are tested in the setup (room1: 51,2; room 2: 43.5; corridor: 41.7). S3 Table of the appendix (B) shows, for the 12 replicates of each group size, the standard deviations linked with the number of majority events (related to S14 Fig). On the S15 Fig, we see that the means of the durations of the majority in each area follow a similar trend in both rooms and are longer than in the corridor. Increasing the size of the group has almost no effect on the durations in the corridor when it has an impact in the rooms, where fish stay longer in majority if the group size increases. However, for 20 zebrafish, durations decrease in all areas. S3 Table of the appendix shows the standard deviations related to the S15 Fig.

FIG. 14: **Means of majority events with a majority of zebrafish in the three areas, for 7 group sizes** for 12 replicates each. To calculate the majority events, we count every time a majority of fish is located in one of the three areas. The red line indicates the number of majority events within the corridor, the blue line shows the number of majority events within the room 1 and the black line in the room 2. The dashed lines distinguish the experiments with one fish from the experiments with groups of fish. For all group sizes except for 20 zebrafish, there are more majority events in the corridor. The majority events are also very similar between room 1 and room 2. The number of majority events is relatively stable for groups of 1, 2, 3, 5 and 7 zebrafish in all three areas.

FIG. 15: **Means of the time spent by a majority of fish in each area.** The results correspond to 12 replicates of 1 hour. The red line represents durations in the corridor, the blue line the durations in the room 1 and the black line the durations in the room 2. The dashed lines distinguish the experiments with one fish from the experiments with groups of fish. We show that the means of the durations are quite similar in rooms 1 and 2. They are shorter in the corridor than in both rooms. Increasing the size of the groups has no effect on the means of the durations in the corridor when it is generally followed by higher durations in both rooms. Finally, the durations strongly decrease when zebrafish are grouped by 20.

TABLE 2: **Mean tracking efficiency.** * means that the experiments are tracked by the idTracker program and that we have the individual identities and the positions of the fish [11].

FIG. 16: **Proportion of fish detected in both rooms.** We show that on average 70% of the fish are detected in the rooms whatever the size of the group. The red square shows the mean and the lighter line the median. We compared the distributions with Kolmogorov-Smirnov tests and found a $p - value < 0.001$ when comparing the distributions of the group sizes of 2 fish versus 20 fish and 3 versus 20. The others comparisons of the distributions where always non significantly different. We set the whiskers between the first and third quartiles.

FIG. 17: **Mean and median number of transitions for different group sizes** when all the fish start to move from a room. The red curve shows *Collective transitions*, the blue curve shows *One-by-one transitions*, the black curve represents the *Collective U-turns* and the magenta (*All transitions*) is the sum of *Collective transitions* and *One-by-one transitions.* The dots show the means and the stars the medians. *One-by-one transitions* occur when the fish transit one by one from one room to the other through the corridor. *Collective transitions* appear when the whole group transit between both rooms through the corridor. *Collective U-turns* occur when the majority of the group was detected successively in one room, in the corridor and back to the previous room. The dashed lines facilitate the lecture. The figure shows that increasing the group sizes makes the number of *Collective U-turns* and *Collective transitions* decrease and the number of *One-by-one transitions* increase. Each point shows the median of 12 values.

TABLE 3: **Standard deviations of the means of majority events, durations, numbers of transition types with a majority of zebrafish in the three sections of the setup**.

FIG. 18: **Probability of occurrence of the rank of exit with the rank of distances from the initiator.** The results correspond to groups of 5 zebrafish (left column) and 10 zebrafish (right column). We counted N = 1456 exits for 12 replicates with 5 zebrafish and N = 277 for 12 replicates with 10 zebrafish. (A) and (B) show the map at the time where the initiator leave the room, (C) and (D) 2 seconds before, (E) and (F) 5 seconds before. As an example, in (A) the probability of occurrence where the second fish leaves the room and has the shortest distance from the initiator is 0.82. As an example, (A) fish with rank of 2 for exit and for distances (closest distance with the initiator) show a probability of 0.82 to be the closest fish to the initiator. This probability decreases to 0.12 for fish with rank of 2 for exit and rank of 3 for distances (the second closest distance with the initiator). Focusing now on (C), 2 seconds before the initiation: the first probability decreases from 0.82 to 0.37 when the second one increases from 0.12 to 0.26. Plots for experiments with 3 and 7 zebrafish are also in the appendix S19 Fig.

FIG. 19: **Probability of occurrence of the rank of exit with the rank of distances from the initiator.** The results correspond to groups of 3 zebrafish (left column) and 7 zebrafish (right column). We counted N = 2195 exits for 12 replicates with 3 zebrafish and N = 1020 for 12 replicates with 7 zebrafish. (A) and (B) show the map at the time where the initiator leave the room, (C) and (D) 2 seconds before, (E) and (F) 5 seconds before.

Bibliography

[1] Séguret, A., Collignon, B., Halloy, J. Strain differences in the collective behaviour of zebrafish (*Danio rerio*) in heterogeneous environment *Royal Society Open Science*, 23:R709, 2016.

[2] Collignon, B., Séguret, A., Halloy, J. A stochastic vision-based model inspired by zebrafish collective behaviour in heterogeneous environments. *Royal Society Open Science*, 2016.

[3] Egan, R. J., Bergner, C. L., Hart, P. C., Cachat, J. M., Canavello, P. R., Elegante, M. F. et al. Understanding behavioral and physiological phenotypes of stress and anxiety in zebrafish. *Behavioural Brain Research*, Volume 205, Issue 1, 14 December 2009, Pages 3844

[4] Chowdhury, D., Schadschneider, A., Nishinari, K. Physics of transport and traffic phenomena in biology: from molecular motors and cells to organisms. *Physics of Life Reviews* Volume 2, Issue 4:318352, 2005.

[5] Burd, M., Aranwela, N. Head-on encounter rates and walking speed of foragers in leaf-cutting ant traffic. *Insectes soc.* 50: 3, 2003.

[6] Herbert-Read, J. E., Krause, S., Morrell, L. J., Schaerf, T. M., Krause, J., Ward, A. J. W. The role of individuality in collective group movement. *Proc R Soc B*, 280: 20122564, 2013.

[7] Magnhagen, C., Bunnefeld, N. Express your personality or go along with the group: what determines the behaviour of shoaling perch? *Proc. R. Soc. B* 276, 33693375, 2009

[8] Burns, A. L., Herbert-Read, J. E., Morrell, L. J., Ward, A. J. Consistency of leadership in shoals of mosquitofish (*Gambusia holbrooki*) in novel and in familiar environments. *PLoS ONE* 7, e36567, 2012

[9] Nakayama, S., Johnstone, R. A., Manica, A. Temperament and hunger interact to determine the emergence of leaders in pairs of foraging fish. *PLoS ONE* 7, e43747, 2012.

[10] King, A. J., Williams, L. J., Mettke-Hofmann, C. The effects of social conformity on Gouldian finch personality. *Animal Behaviour*, Volume 99, January 2015, Pages 2531, 2015.

[11] Pérez-Escudero, A., Vicente-Page, J., Hinz, R. C., Arganda, S., De Polavieja, G. G. idTracker: tracking individuals in a group by automatic identification of unmarked animals *Nature methods*, 11(7):743–748, 2014.
