# Supplementary figures and images for: Loose social organisation of AB strain zebrafish groups in a two-patch environment
Source: PLoS One. 2019 Feb 8;14(2):e0206193. doi: 10.1371/journal.pone.0206193 (PMC6368274; doi:10.1371/journal.pone.0206193)

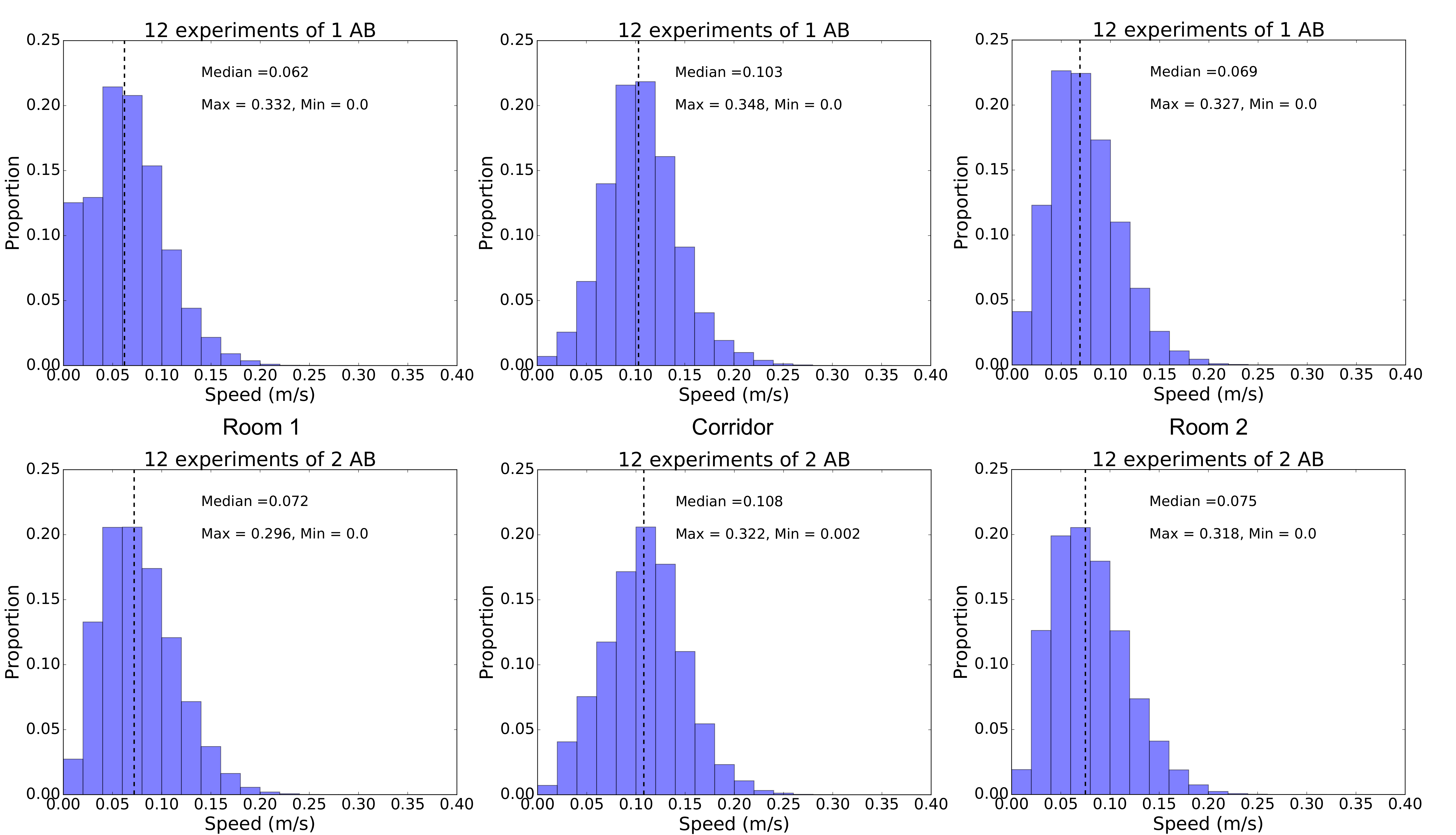

Supplement: S1 Fig — (TIFF) [file pone.0206193.s002.tiff]

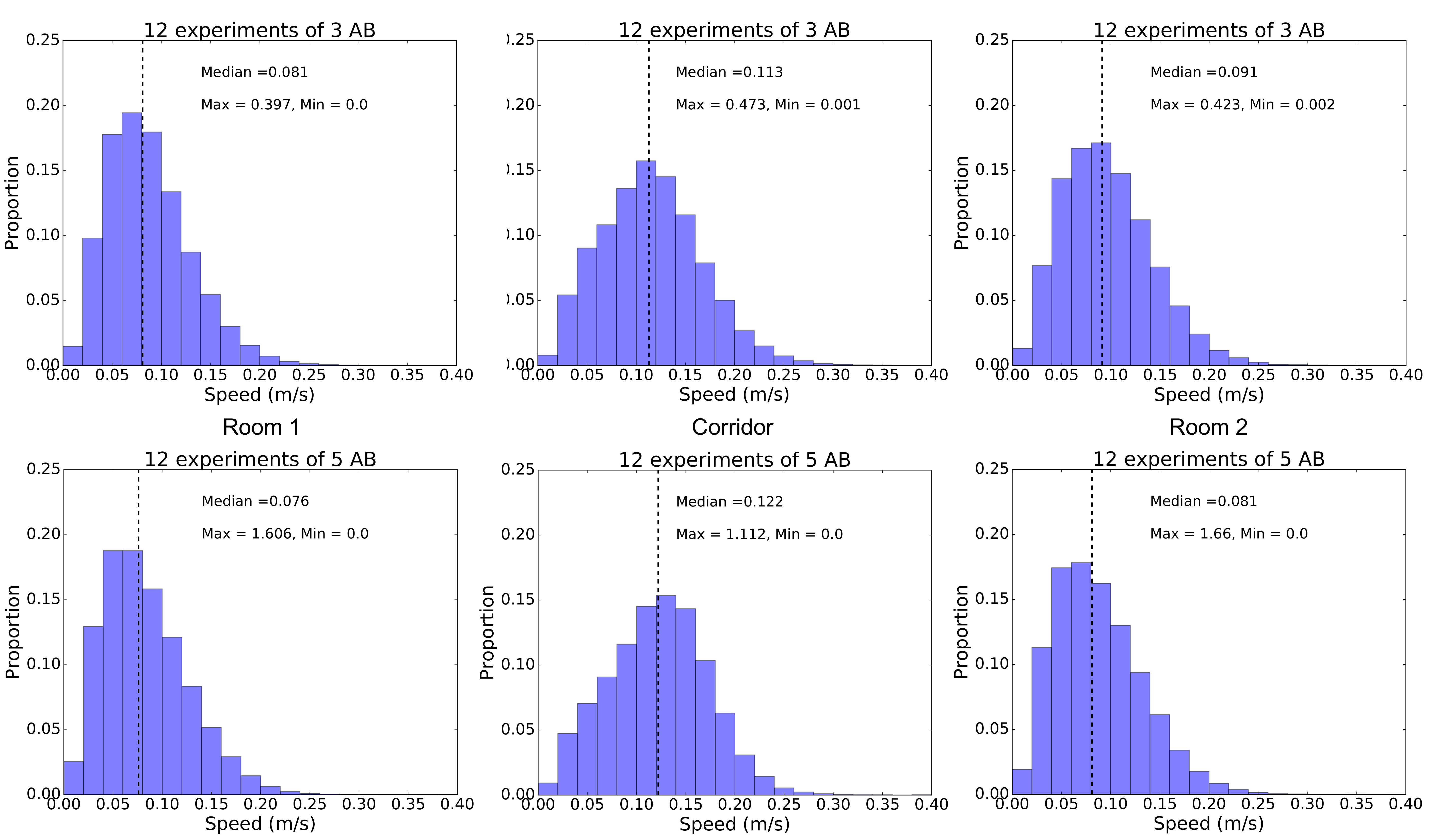

Supplement: S2 Fig — Groups of 3 and 5 AB zebrafish. (TIFF) [file pone.0206193.s003.tiff]

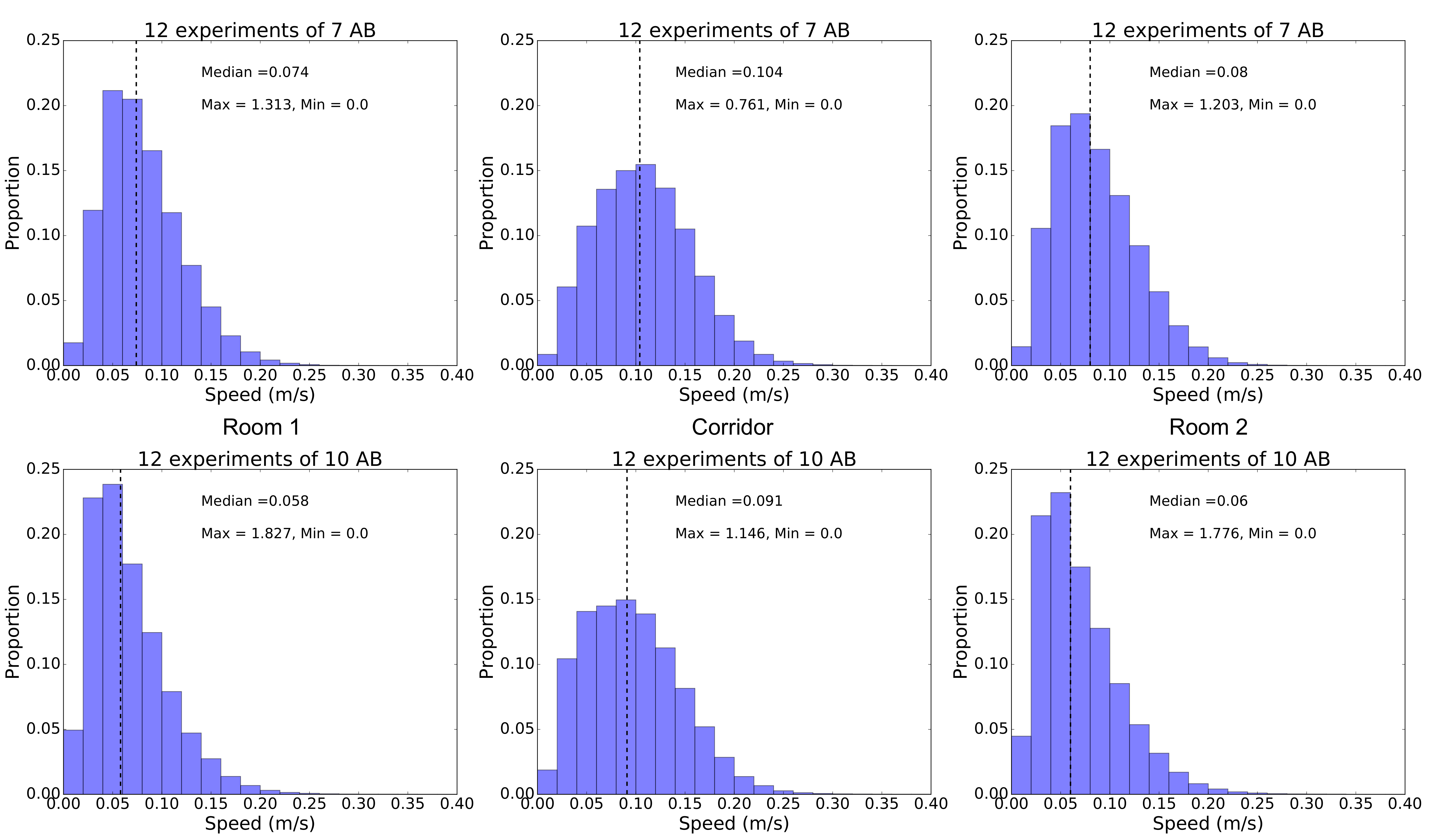

Supplement: S3 Fig — Groups of 7 and 10 AB zebrafish. (TIFF) [file pone.0206193.s004.tiff]

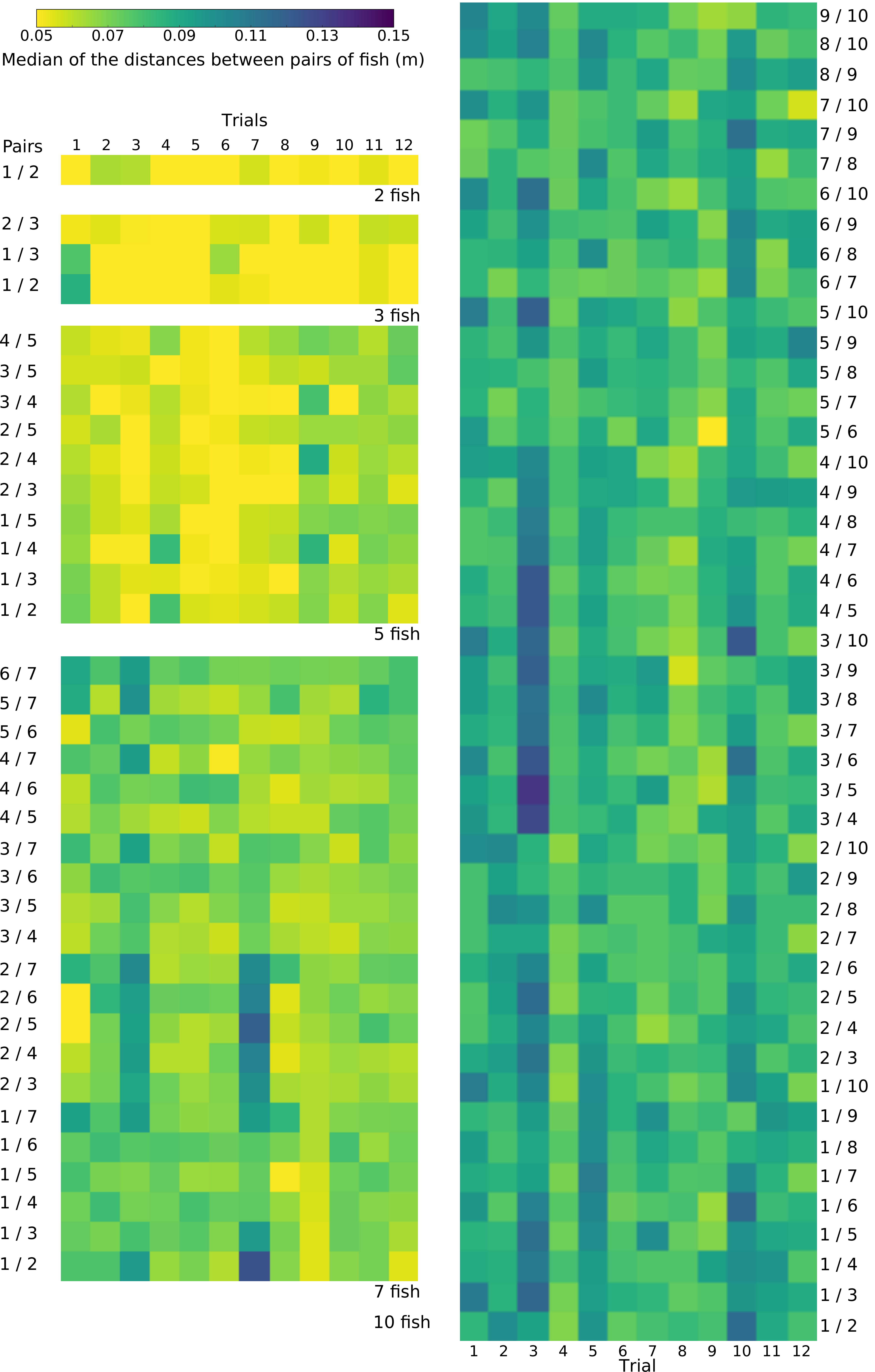

Supplement: S4 Fig — (TIFF) [file pone.0206193.s005.tiff]

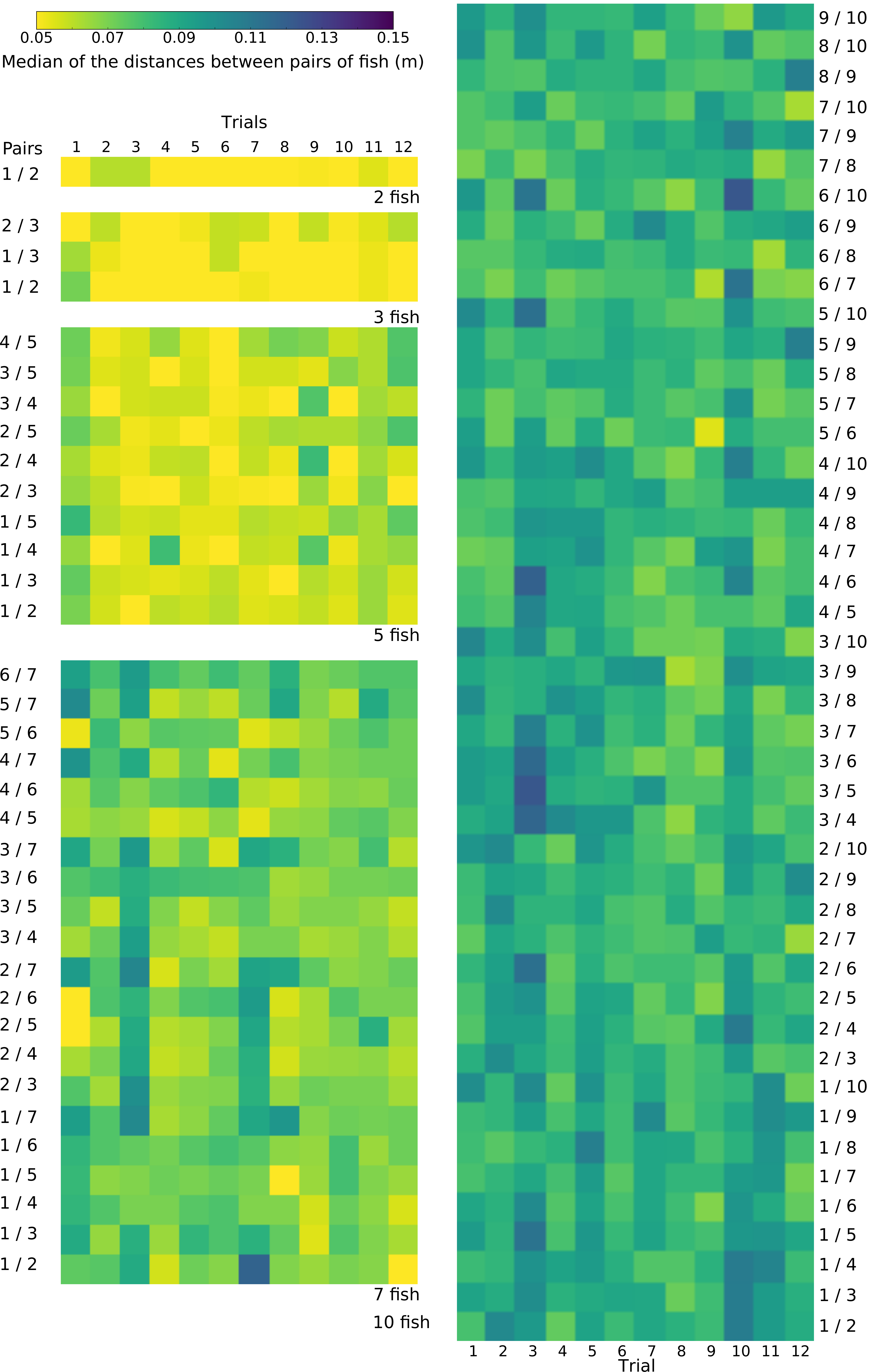

Supplement: S5 Fig — (TIFF) [file pone.0206193.s006.tiff]

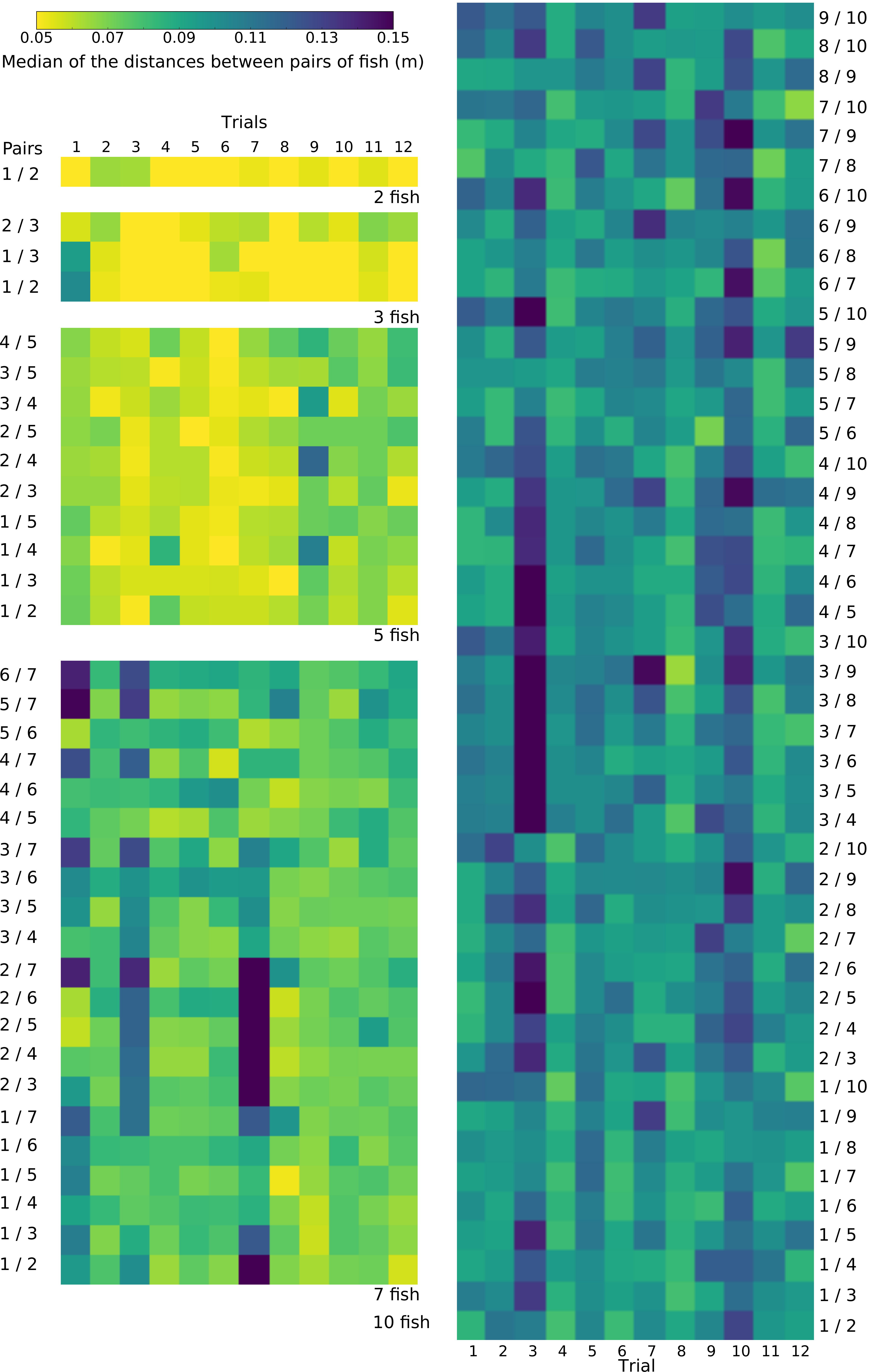

Supplement: S6 Fig — (TIFF) [file pone.0206193.s007.tiff]

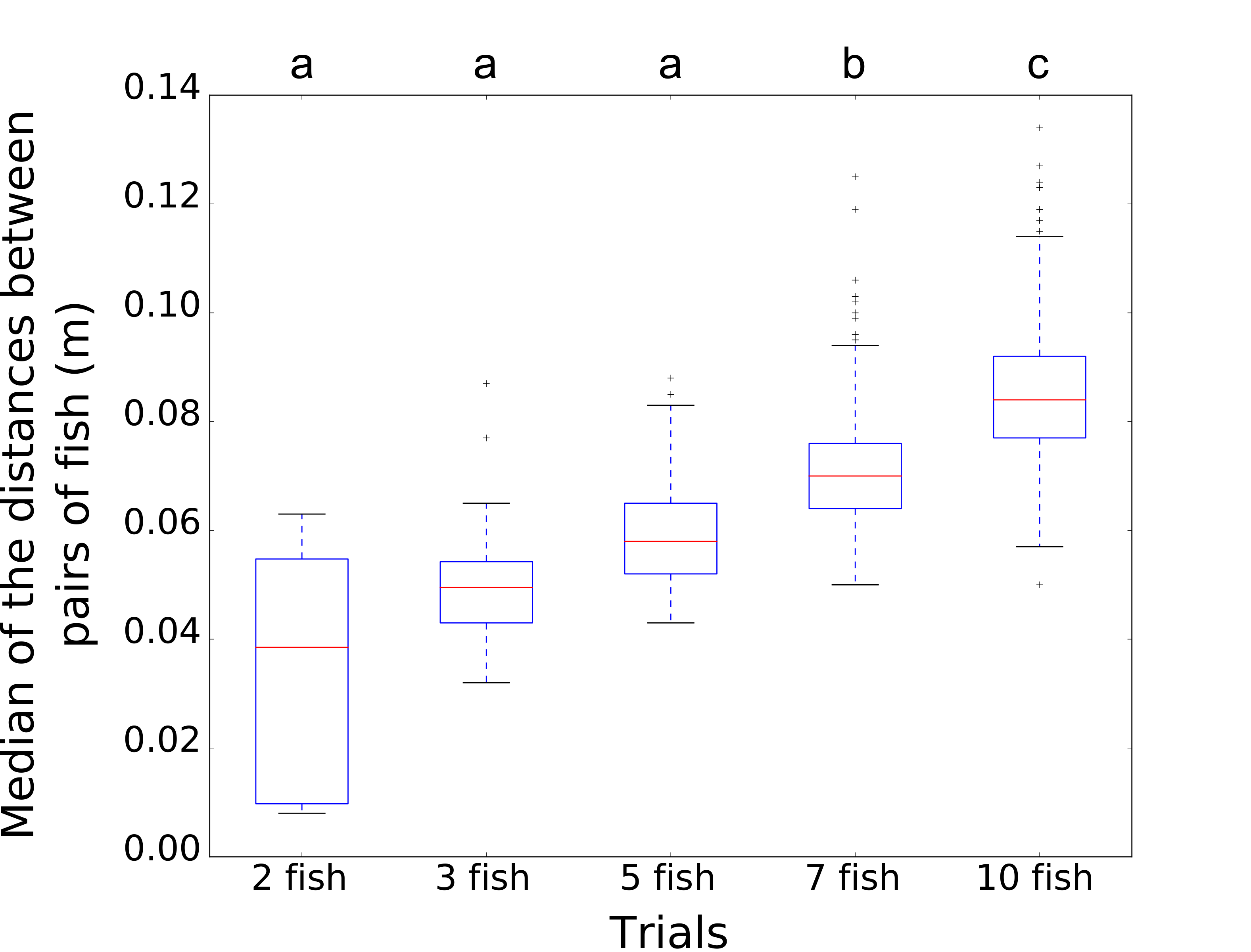

Supplement: S7 Fig — (TIFF) [file pone.0206193.s008.tiff]

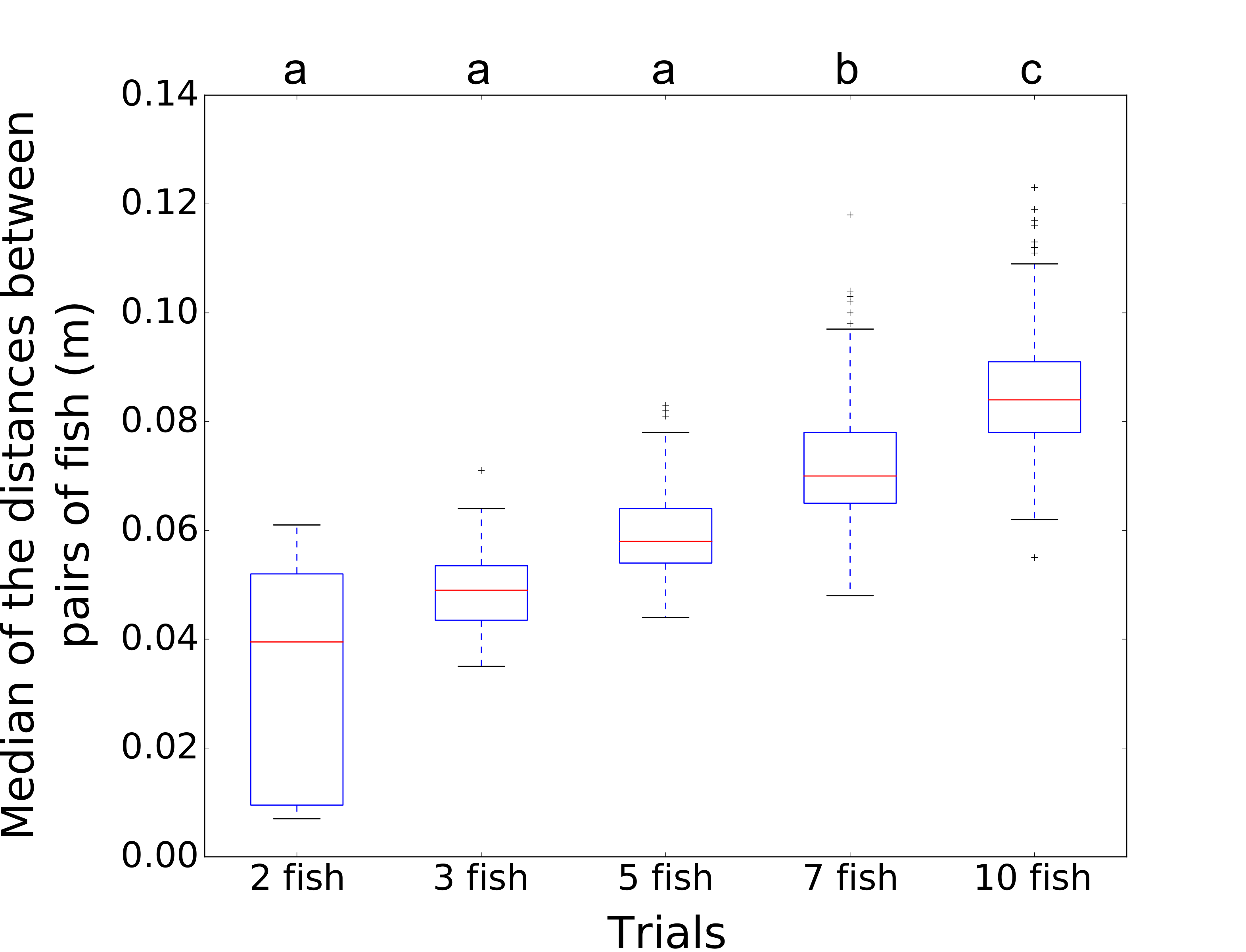

Supplement: S8 Fig — (TIFF) [file pone.0206193.s009.tiff]

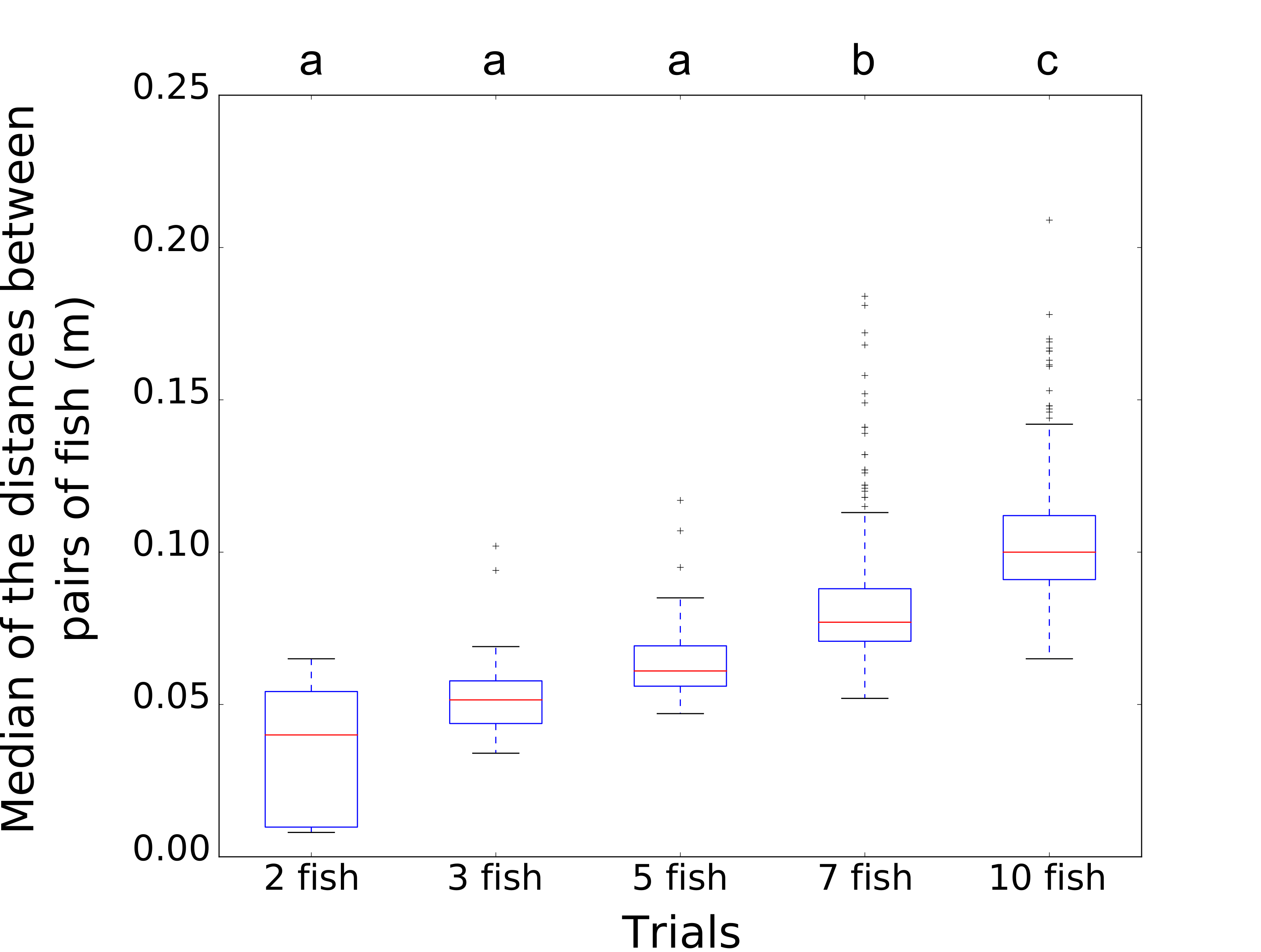

Supplement: S9 Fig — (TIFF) [file pone.0206193.s010.tiff]

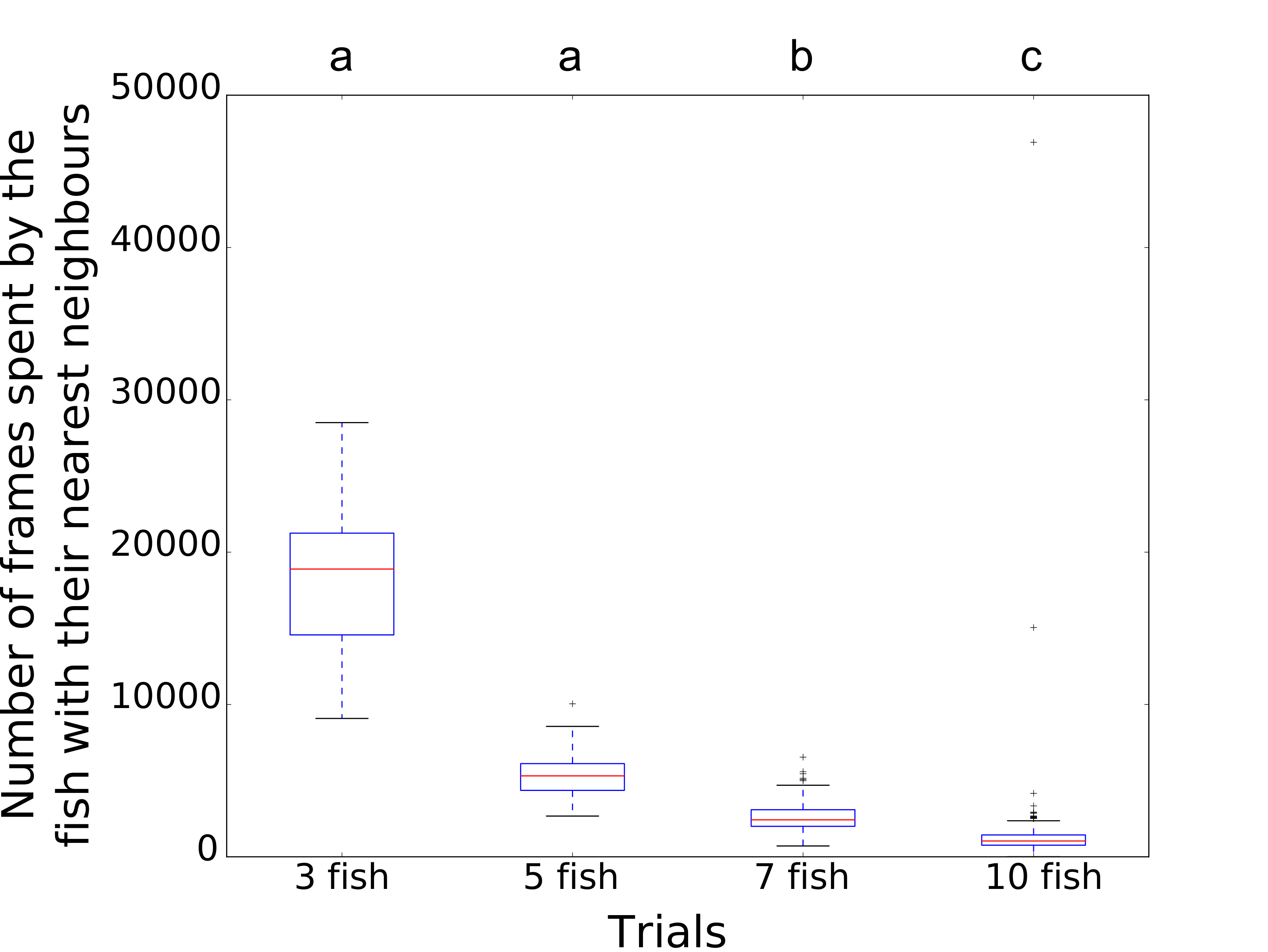

Supplement: S10 Fig — (TIFF) [file pone.0206193.s011.tiff]

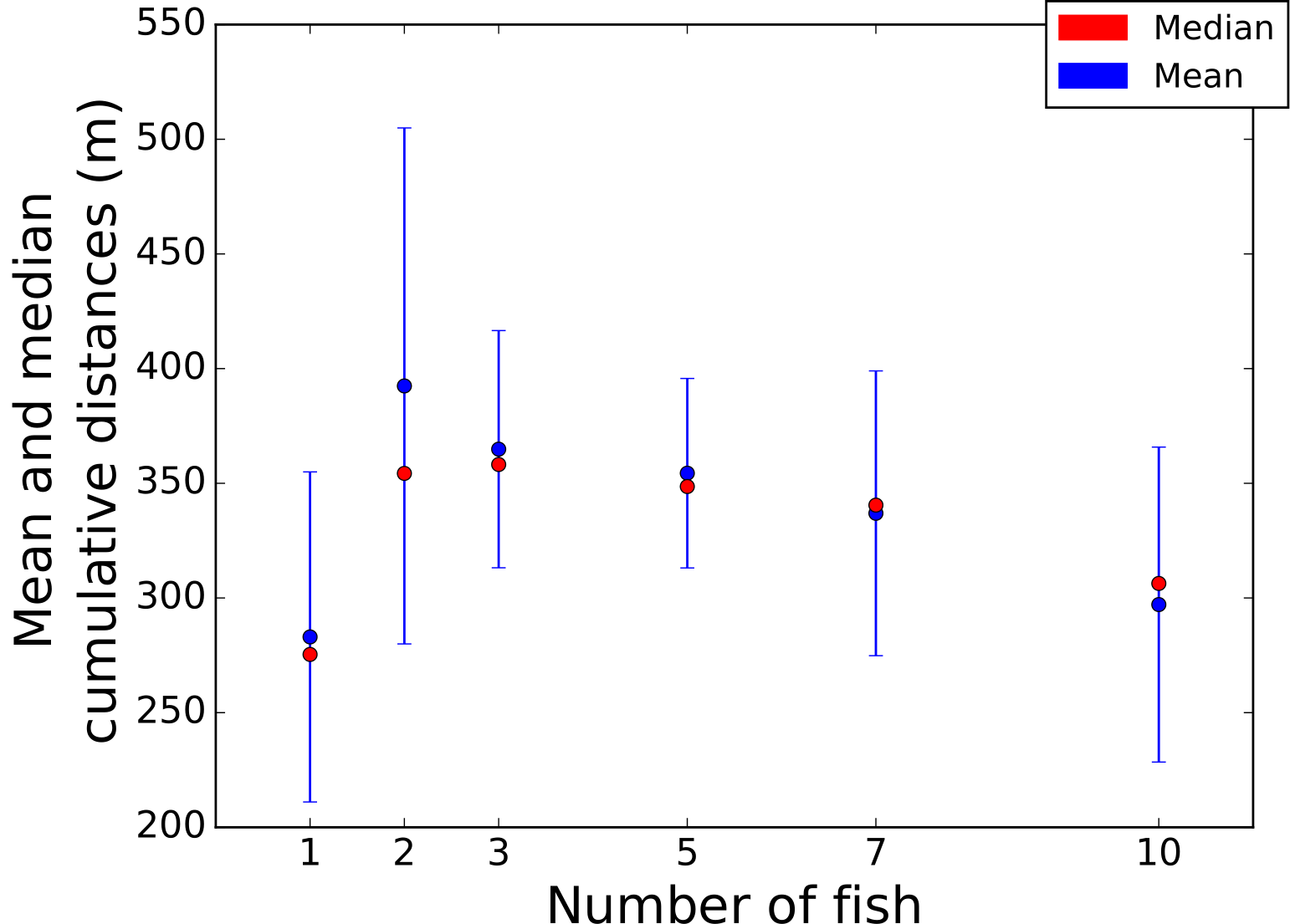

Supplement: S11 Fig — (TIFF) [file pone.0206193.s012.tiff]

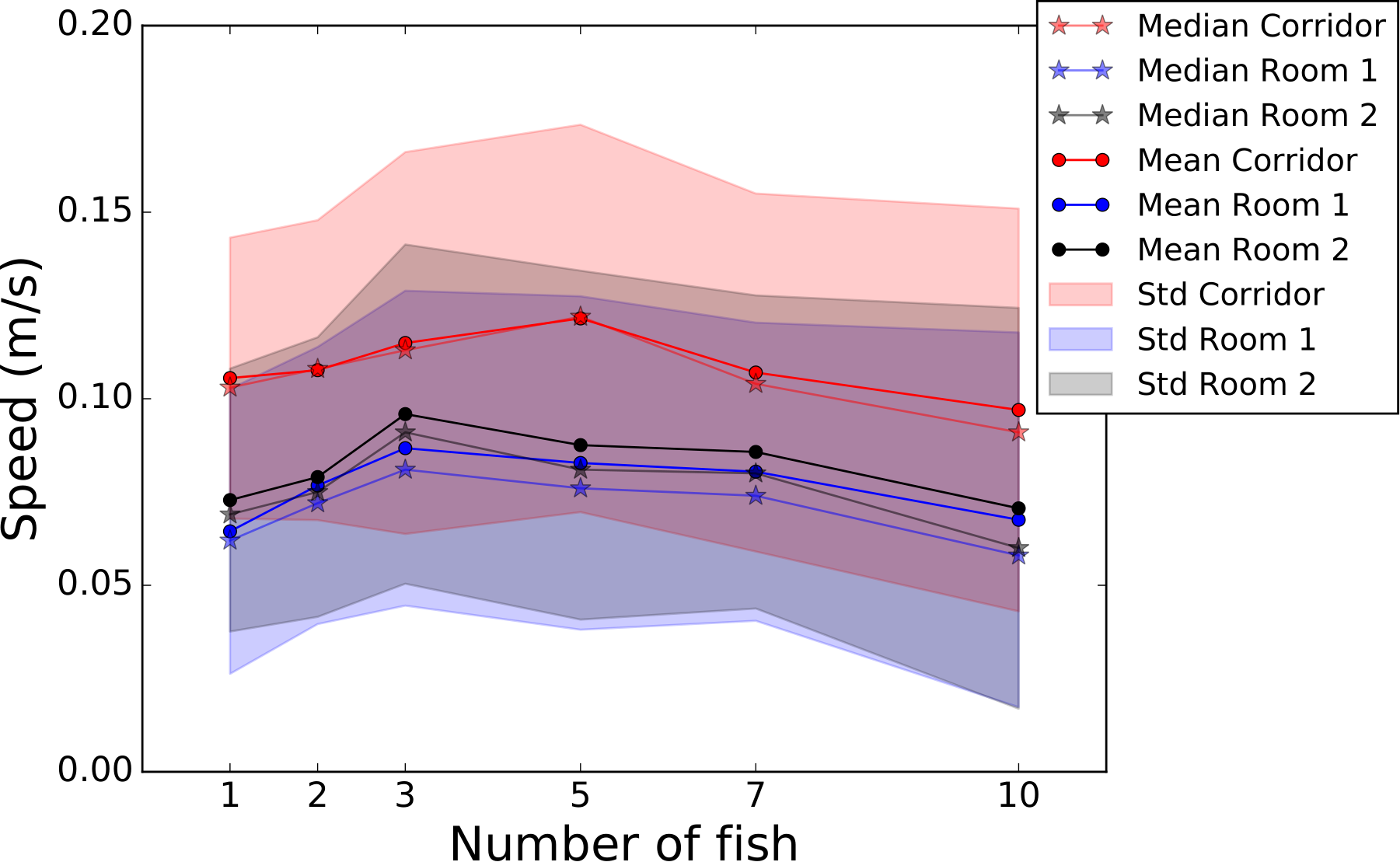

Supplement: S12 Fig — (TIFF) [file pone.0206193.s013.tiff]

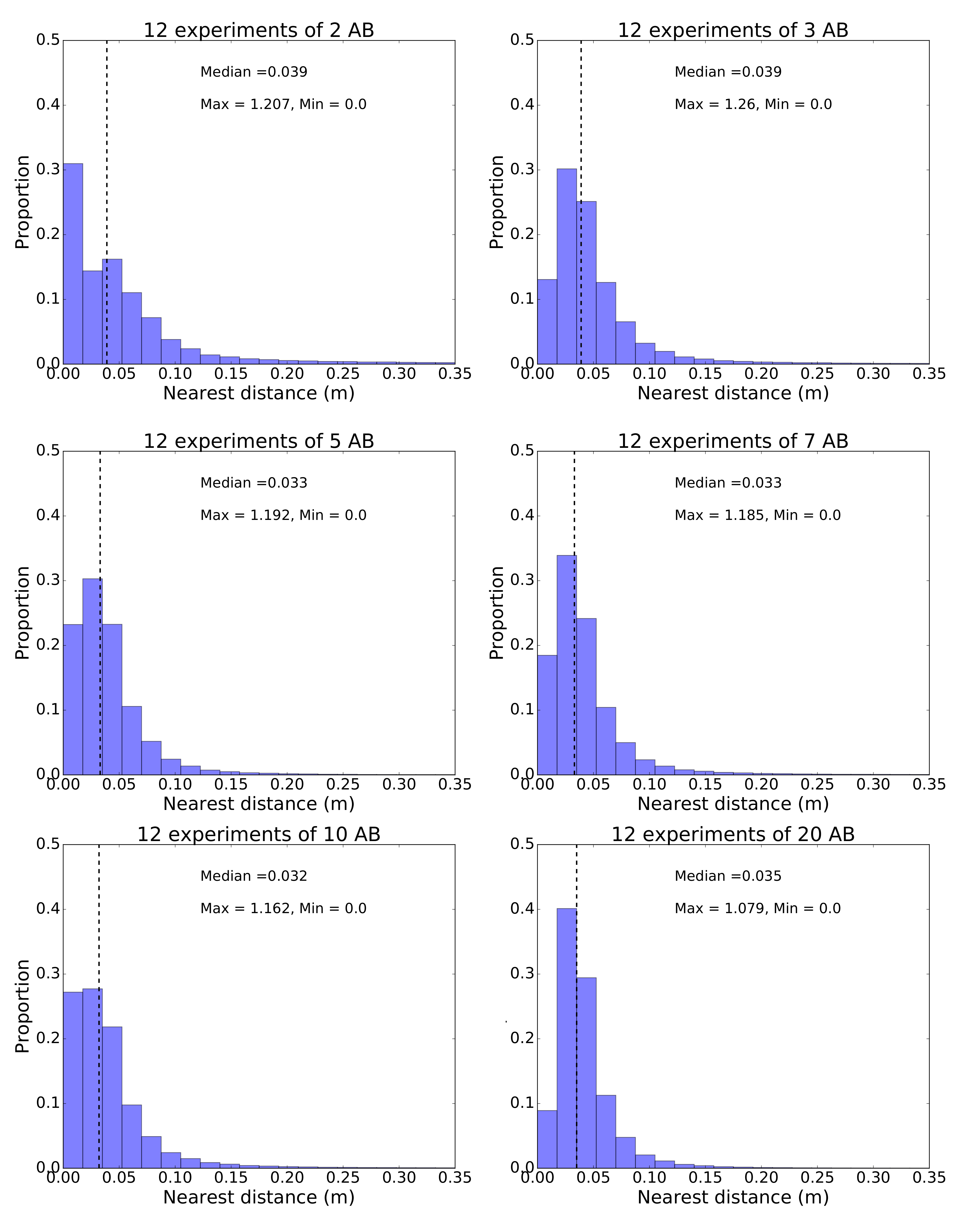

Supplement: S13 Fig — (TIFF) [file pone.0206193.s014.tiff]

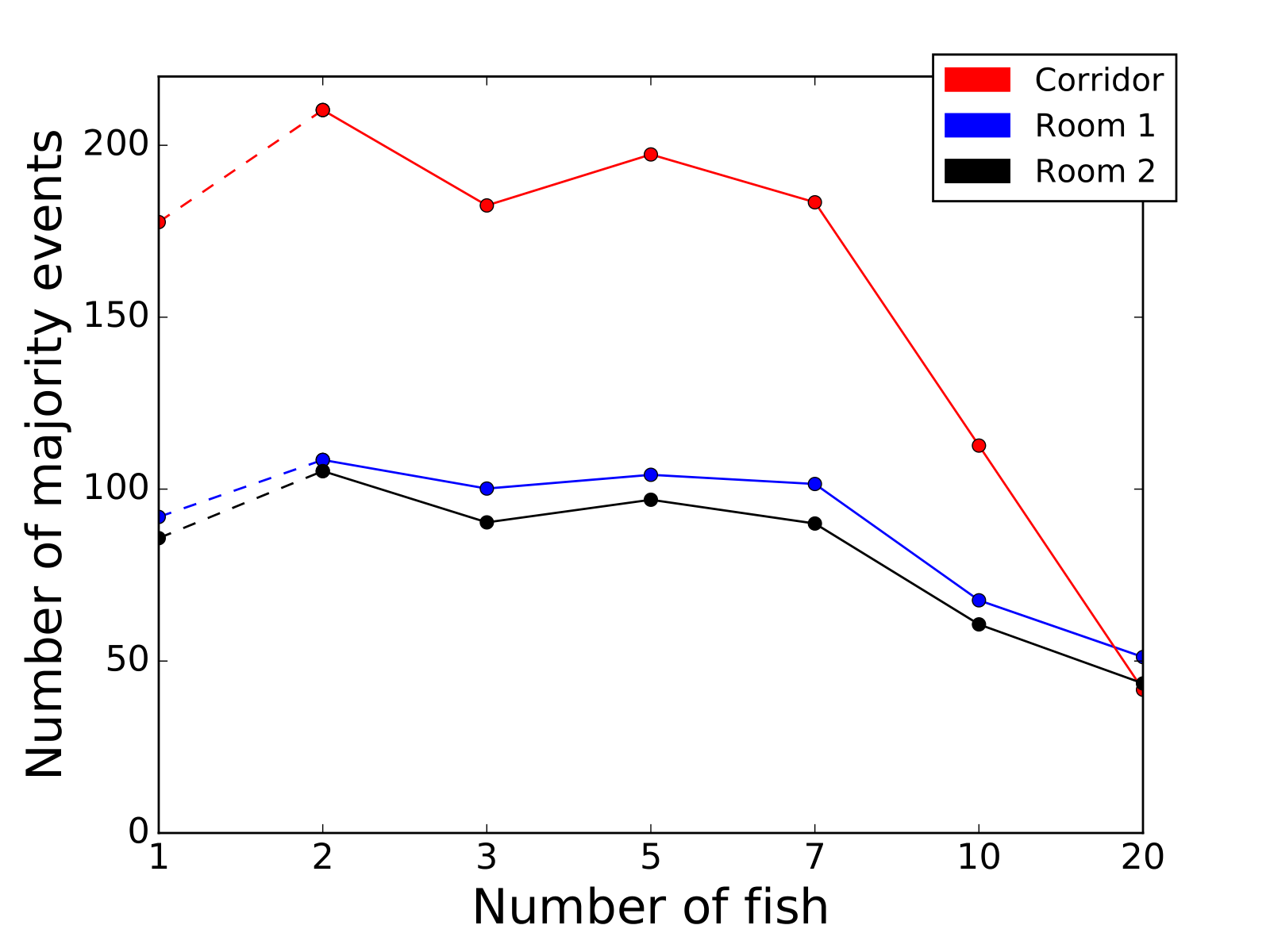

Supplement: S14 Fig — (TIFF) [file pone.0206193.s015.tiff]

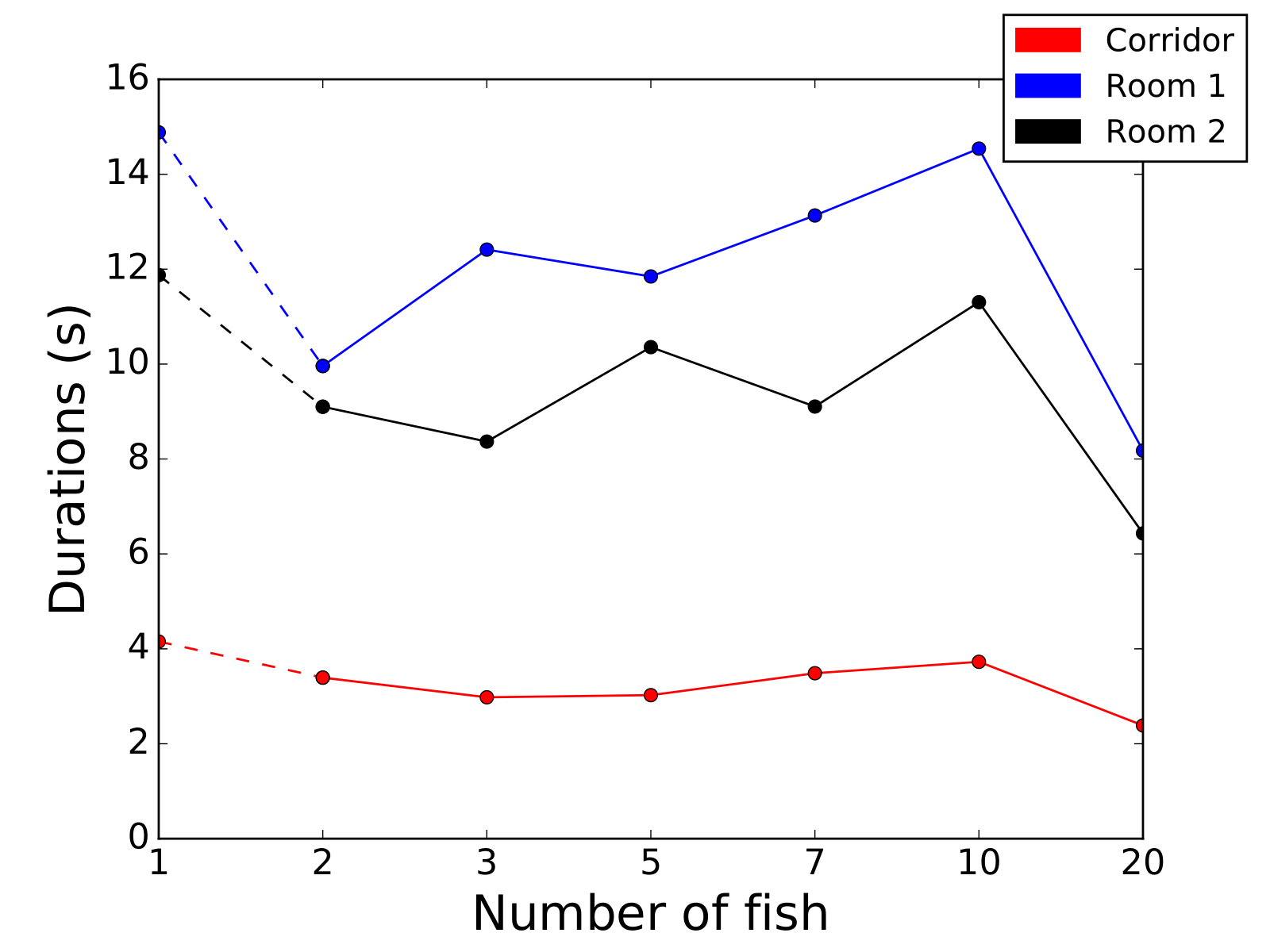

Supplement: S15 Fig — (TIFF) [file pone.0206193.s016.tiff]

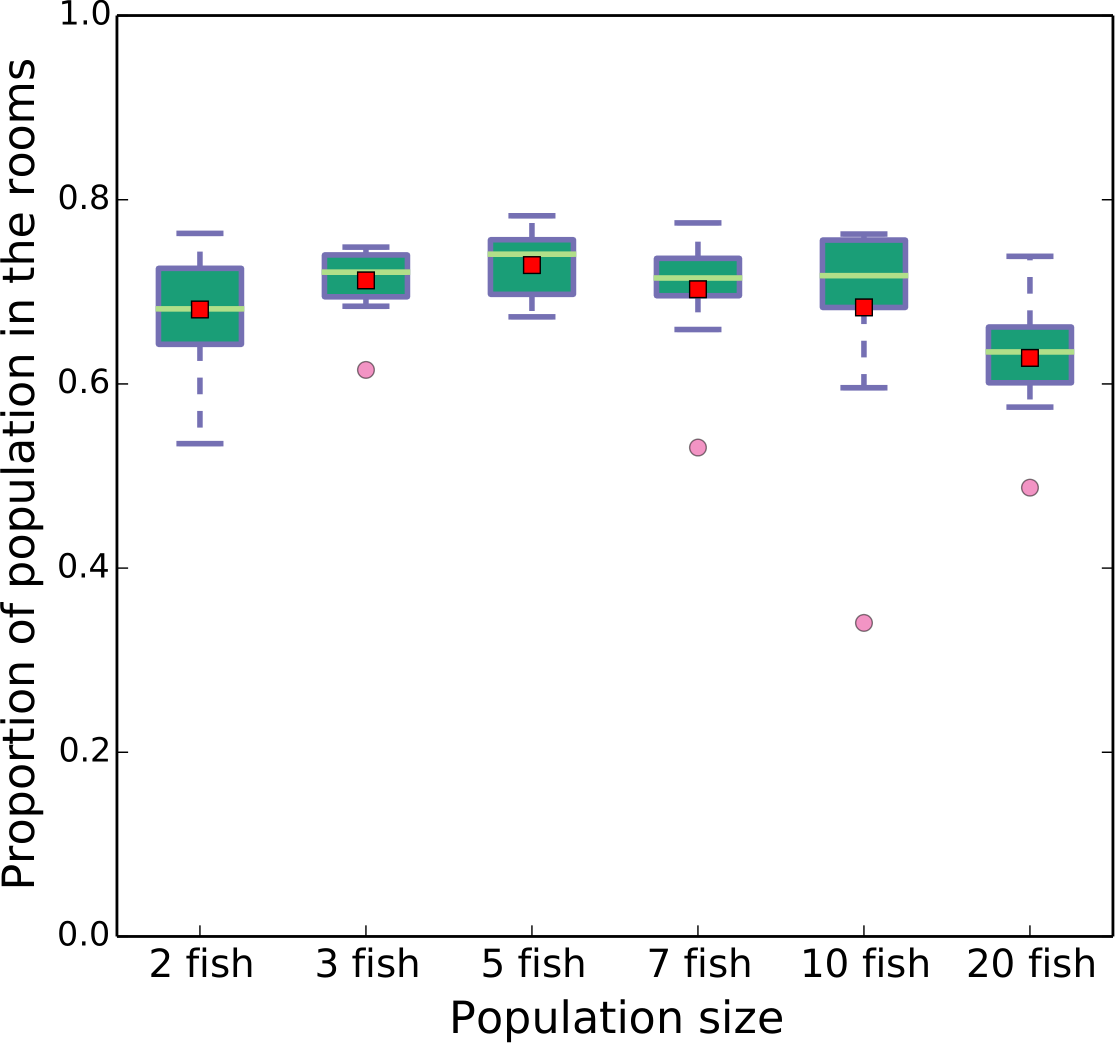

Supplement: S16 Fig — (TIFF) [file pone.0206193.s017.tiff]

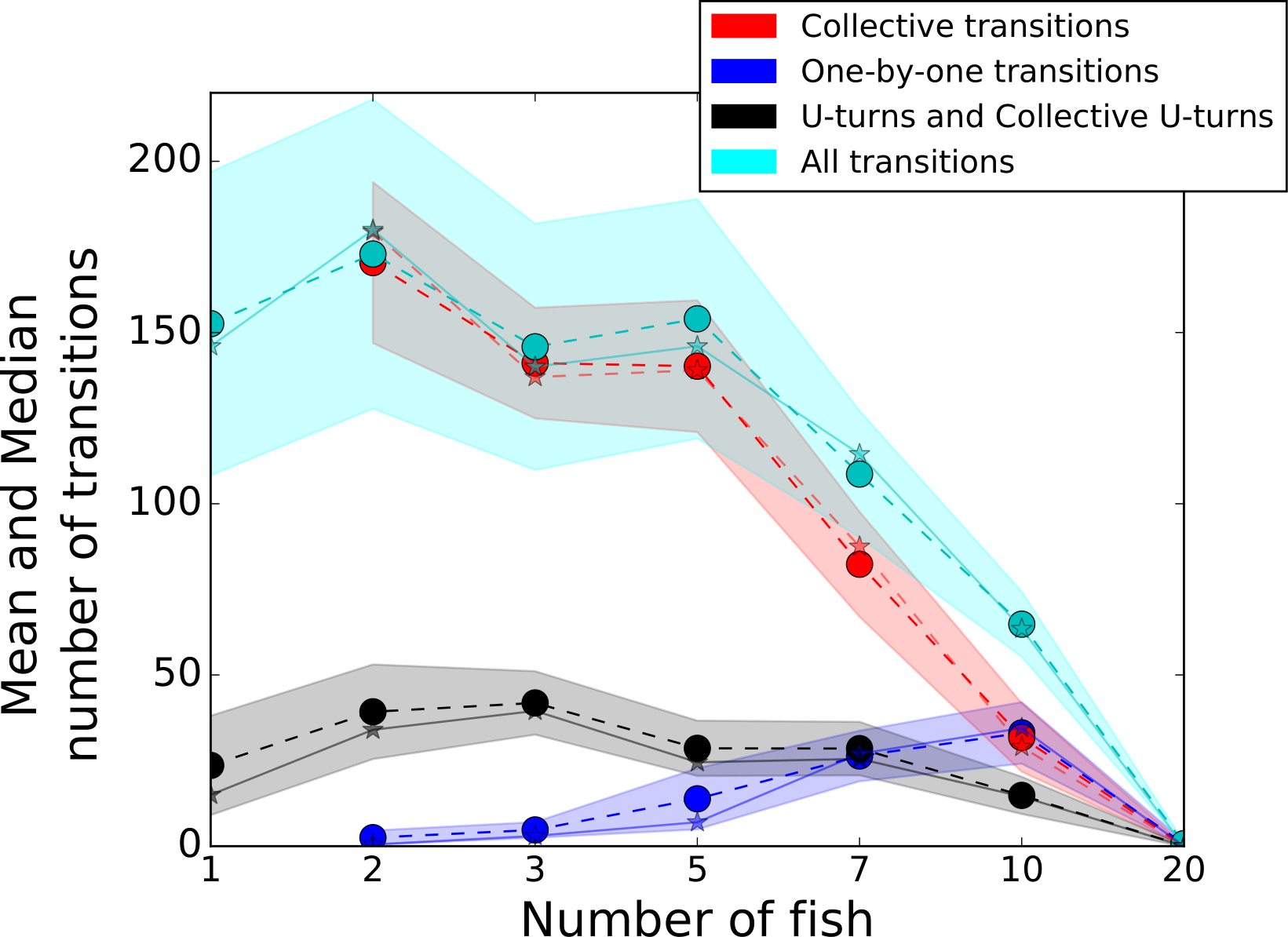

Supplement: S17 Fig — (TIFF) [file pone.0206193.s018.tiff]

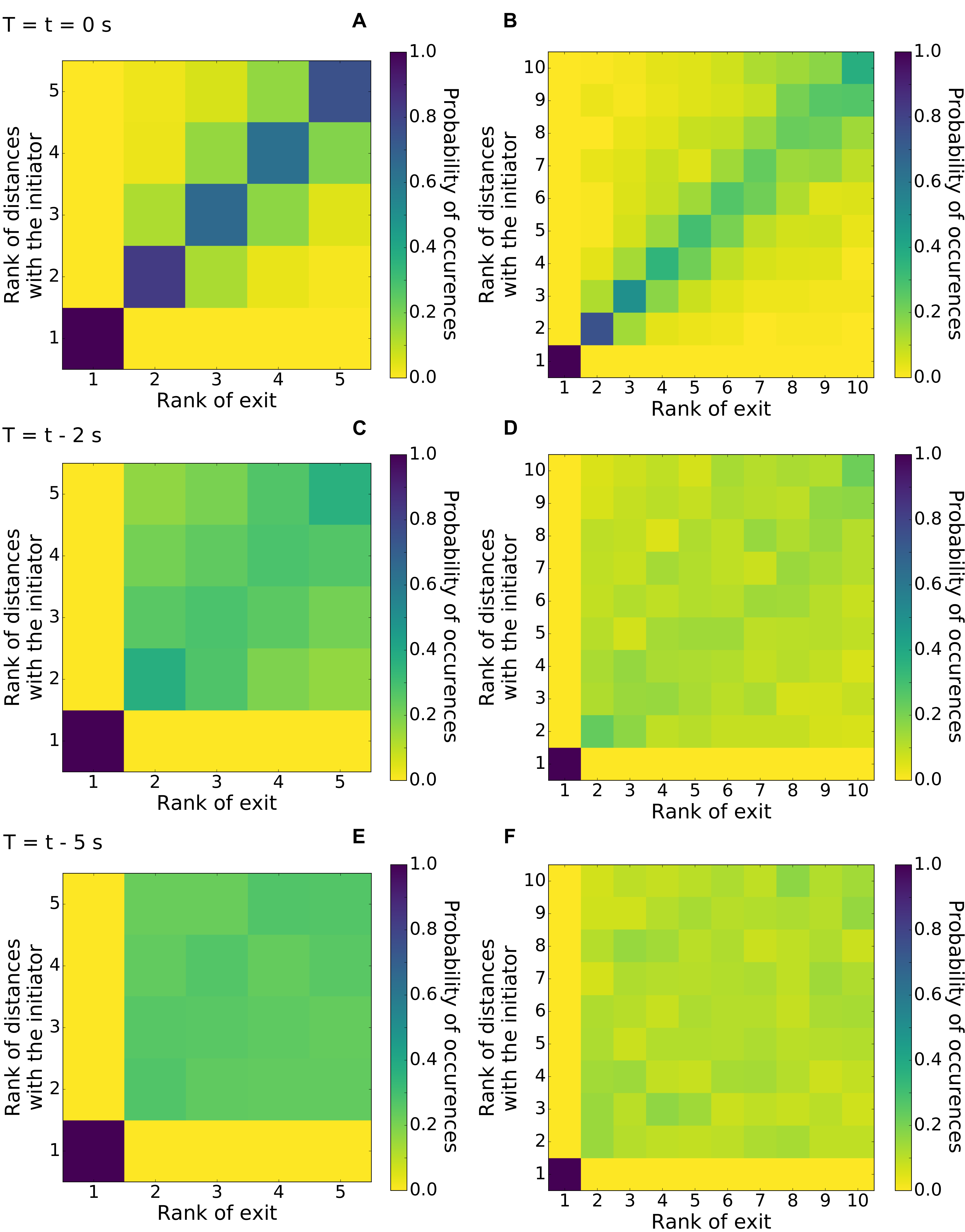

Supplement: S18 Fig — (TIFF) [file pone.0206193.s019.tiff]

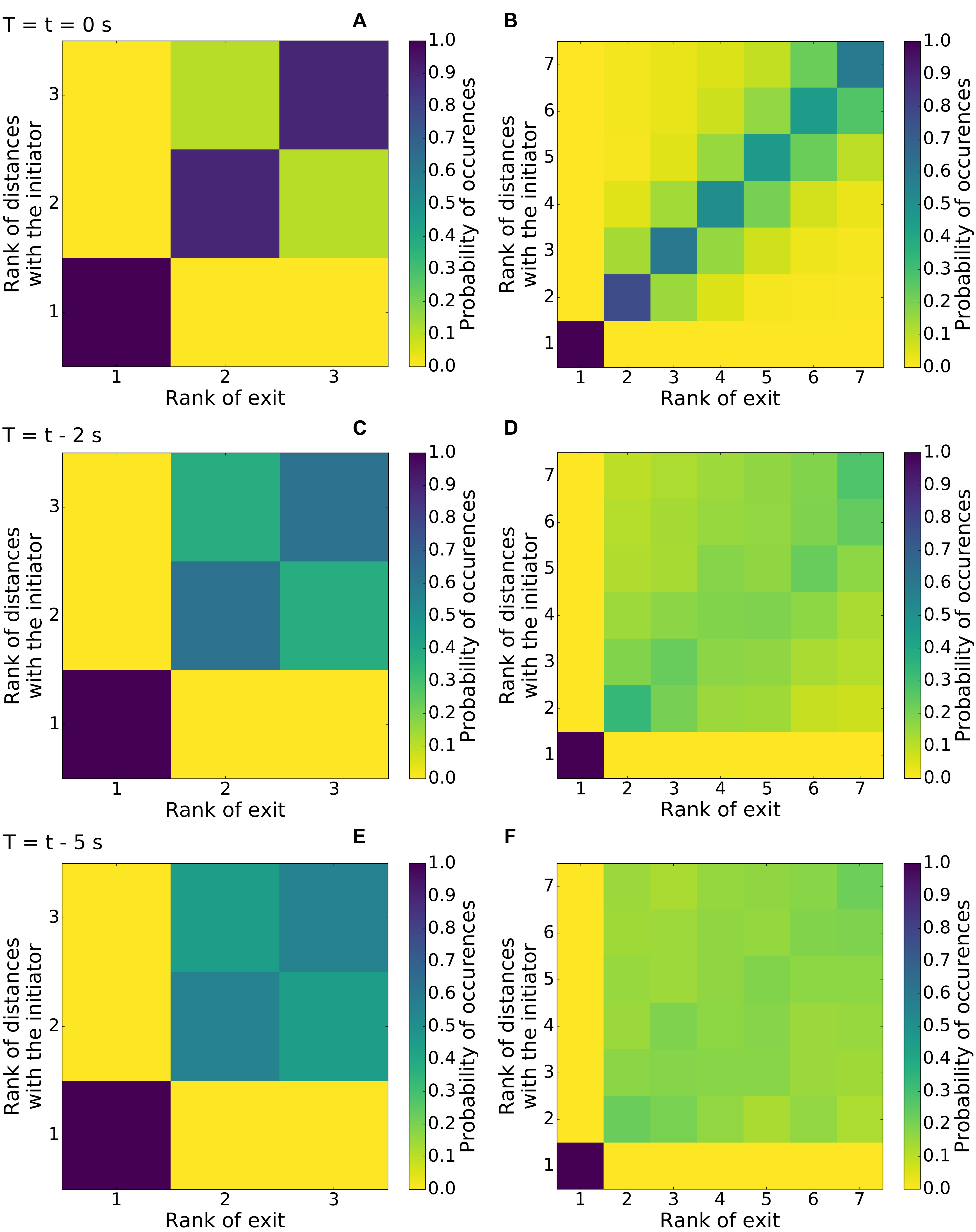

Supplement: S19 Fig — (TIFF) [file pone.0206193.s020.tiff]
